# Supplementary material for: A weighted two-stage sequence alignment framework to identify motifs from ChIP-exo data
Source: Patterns (N Y). 2024 Feb 2;5(3):100927. doi: 10.1016/j.patter.2024.100927 (PMC10935504; doi:10.1016/j.patter.2024.100927)
Supplement: Document S1. Notes S1–S5 [file mmc1.pdf]

Patterns, Volume *5*

Supplemental information

A weighted two-stage sequence alignment framework to identify motifs from ChIP-exo data

Yang Li, Yizhong Wang, Cankun Wang, Anjun Ma, Qin Ma, and Bingqiang Liu

1. Note S1: Parameters for benchmarking algorithms

We execute the seven designated motif discovery algorithms using specific options, with values contingent upon the individual experiment. Version numbers for the algorithms are denoted immediately after their respective names.

- BoBro v.2.0:
  ```
  BoBro -i <primary> -l 14 -o 100
  ```
  where <primary> is the file of input sequences.
- Homer v.4.11:
  ```
  homer2 denovo -i <primary> -b <control> -S 100 -len 10 > <outdir>
  ```
  where <outdir> is the directory where Homer will write its results and <control> is the file of background sequences.
- MEME v.5.5.4:
  ```
  meme <primary> -dna -revcomp -minw 8 -maxw 50 -nmotifs 100
  ```
- MFMD v.1.0:
  ```
  java -jar mfmd.jar <primary> 22 0.005
  ```
- PEnGmotif v.1.0.1:
  ```
  PEnGmotif <primary> -o <outdir> --background-sequences <control>
  ```
- STREME v.5.5.4:
  ```
  streme -p <primary> -n <control> -dna -minw 8 -maxw 15 -kmer 3 -text
  ```
- TESA v.1.0:
  ```
  TESA -i <primary> -l 14 -n 1 -c 0.8 -u 0.9 -o 100
  ```
- XXmotif v.1.6:
  ```
  XXmotif <outdir> <primary> --revcomp
  ```

2. Note S2: Alignment of TESA-cov and reference motifs

In each panel, the top two lines display the HOCOMOCO[1] motif used for experimental evaluation. The sequence logos depict the TESA motif (bottom) juxtaposed with the reference motif (top). Above these aligned logos, the Tomtom P-value represents the accuracy of the motif identified by TESA, with a lower value indicating greater similarity to the reference motif.

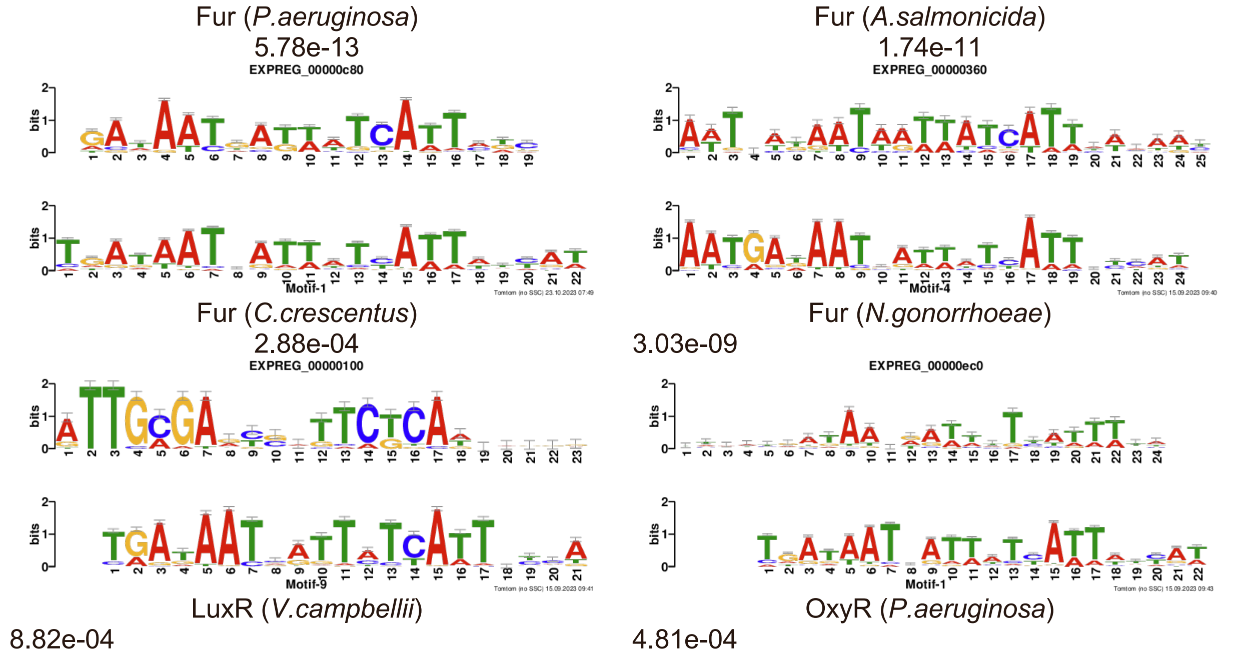

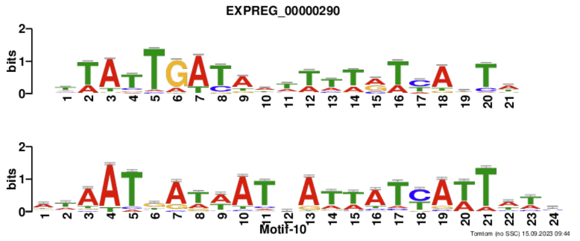

NikR (*H.pylori*)
1.34e-03

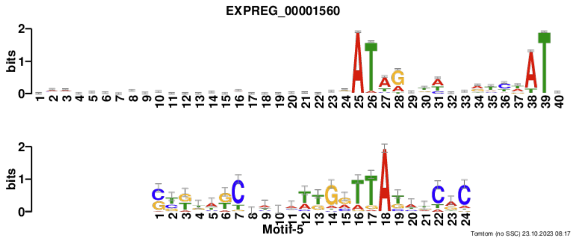

GlxR (*C.glutamicum*)
5.87e-06

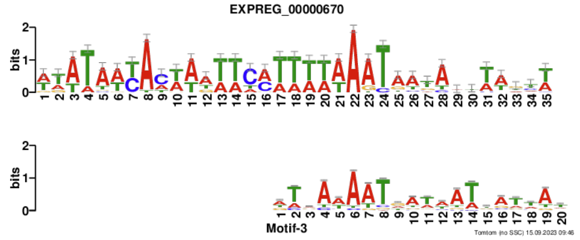

Fur (*P.syringae*)
4.35e-07

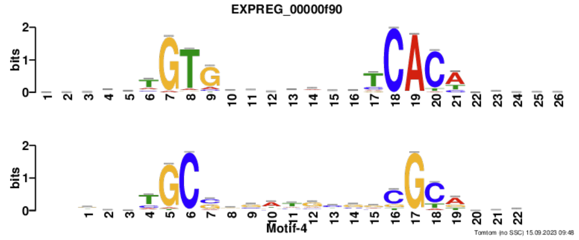

CodY (*L.lactis*)
2.06e-05

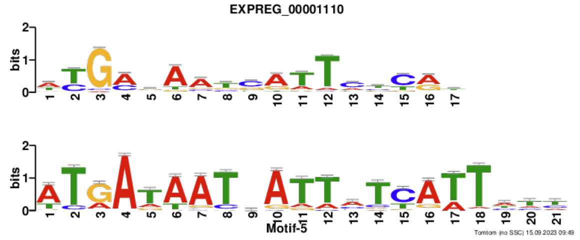

ArgR (*P.aeruginosa*)
8.41e-04

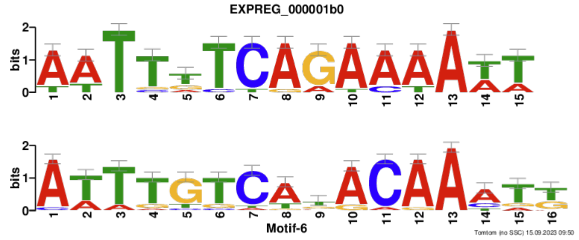

Fur (*B.subtilis*)
8.66e-12

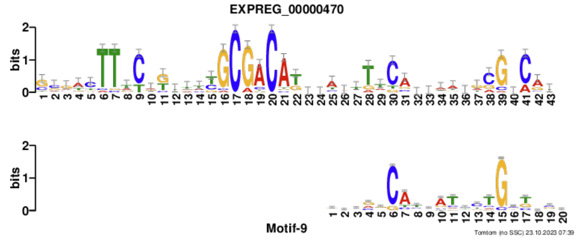

PerR (*B.subtilis*)
1.83e-05

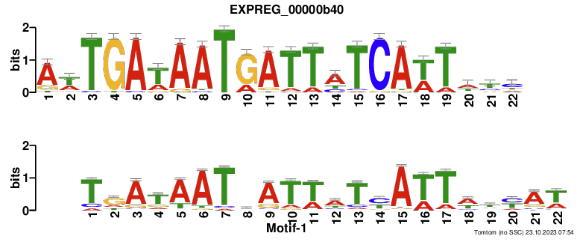

AmrZ (*P.aeruginosa*)
6.04e-04

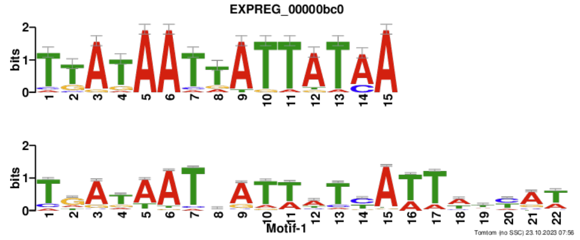

H-NS (*V.cholerae*)
7.33e-06

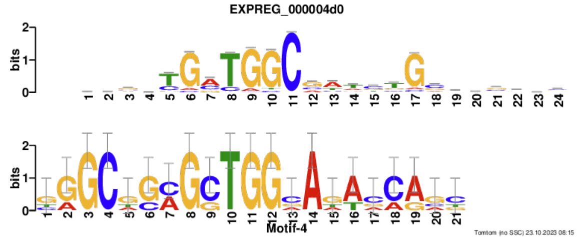

IHF (*P.putida*)
5.27e-06

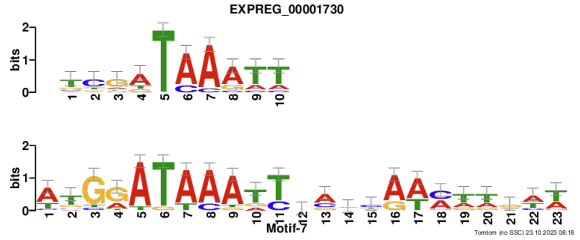

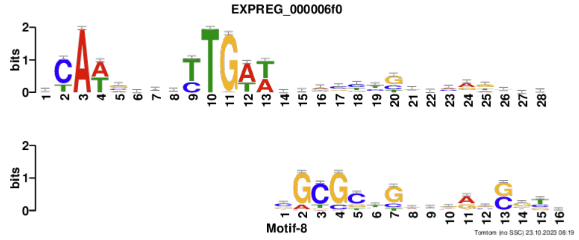

Vfr (*P.aeruginosa*)
1.56e-03

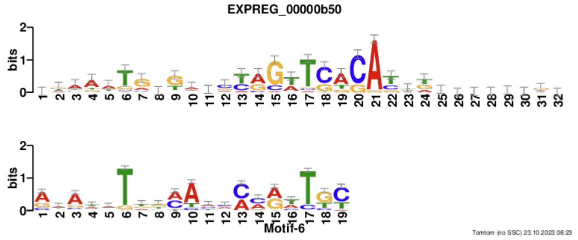

PvdS (*P.aeruginosa*)
2.50e-04

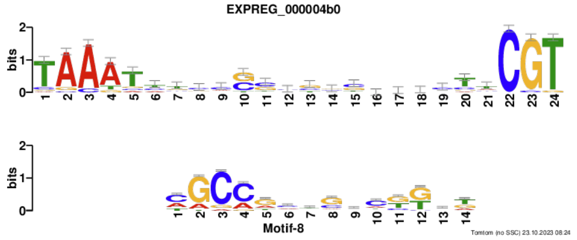

VqsM (*P.aeruginosa*)
4.57e-04

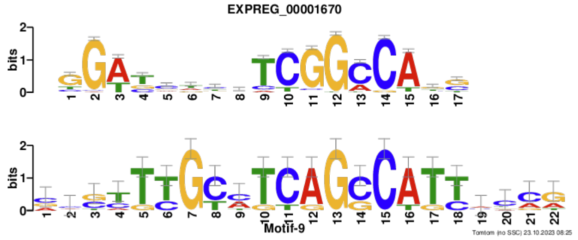

LexA (*M.tuberculosis*)
5.55e-04

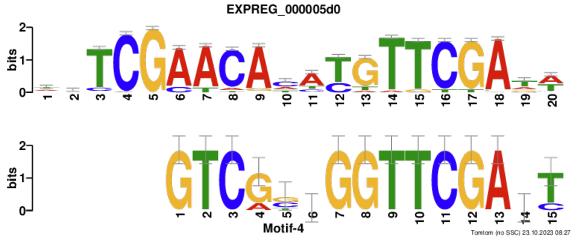

DosR (*M.tuberculosis*)
7.47e-04

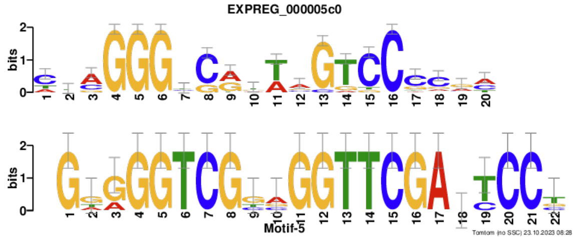

HrpX (*X.oryzae*)
2.63e-04

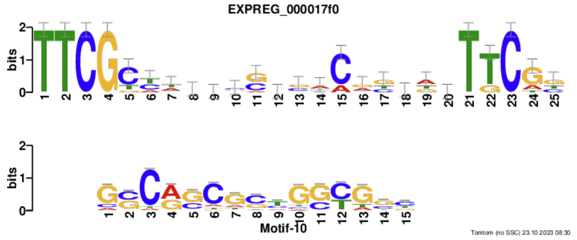

EspR (*M.tuberculosis*)
4.72e-04

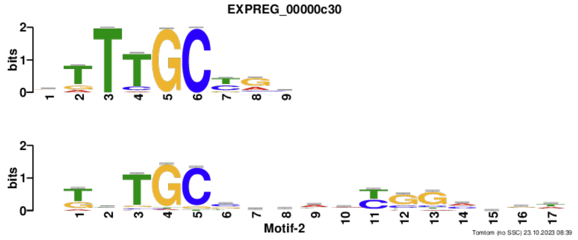

MogR (*L.monocytogenes*)
4.39e-04

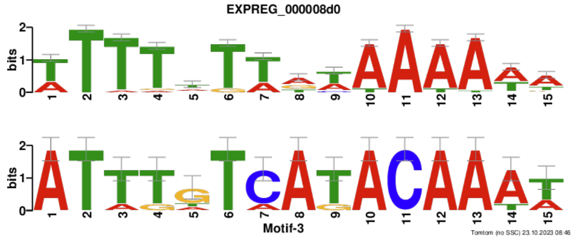

OmpR (*Y.pestis*)
5.18e-04

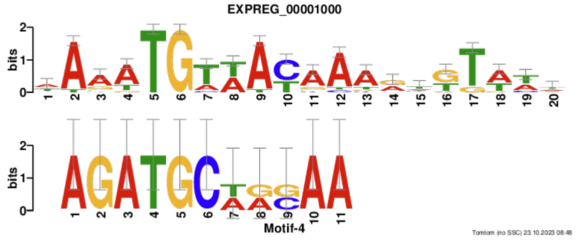

ZN502 (*H. sapiens*)
7.76e-10

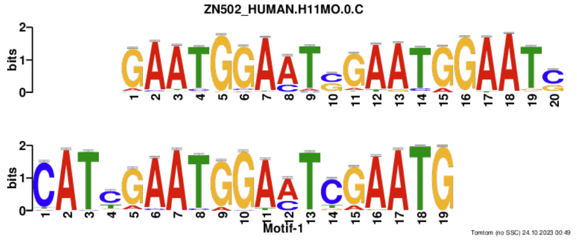

SMCA5 (*H. sapiens*)
4.89e-08

ZN341 (*H. sapiens*)
2.98e-09

SMCA5_HUMAN.H11MO.0.C

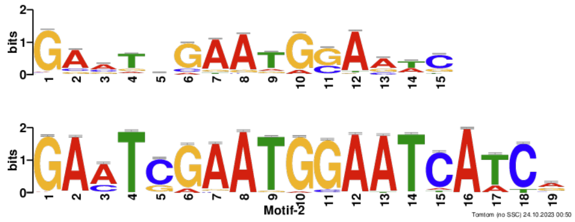

PO2F1 (*H. sapiens*)
1.28e-05

PO2F1_HUMAN.H11MO.0.C

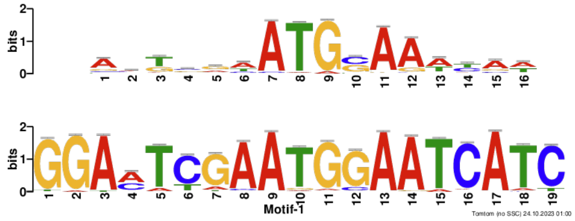

TBX15 (*H. sapiens*)
5.89e-09

TBX15_HUMAN.H11MO.0.D

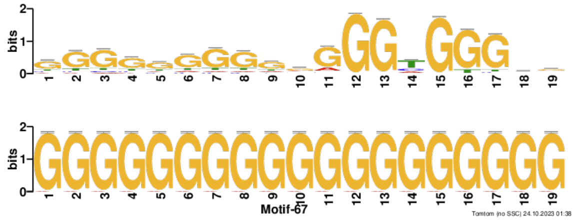

KLF16 (*H. sapiens*)
5.94e-08

KLF16_HUMAN.H11MO.0.D

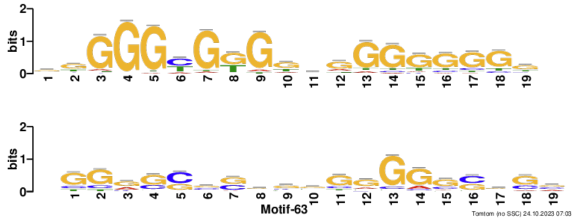

ZNF8 (*H. sapiens*)
4.75e-05

ZNF8_HUMAN.H11MO.0.C

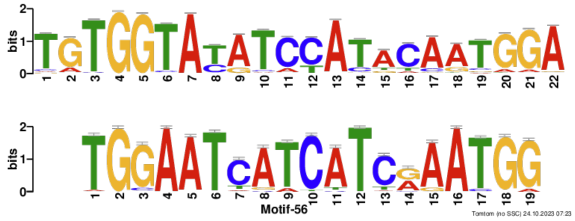

ZN394 (*H. sapiens*)
5.03e-05

ZN394_HUMAN.H11MO.1.D

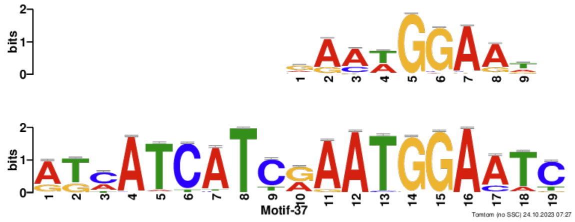

ZN341_HUMAN.H11MO.0.C

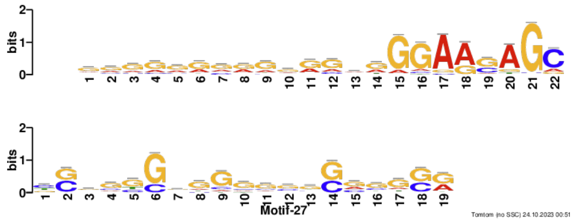

CENPB (*H. sapiens*)
2.08e-07

CENPB_HUMAN.H11MO.0.D

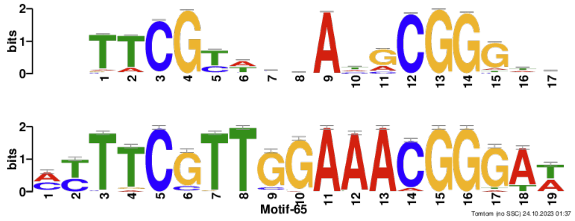

ATF2 (*H. sapiens*)
7.69e-05

ATF2_HUMAN.H11MO.1.B

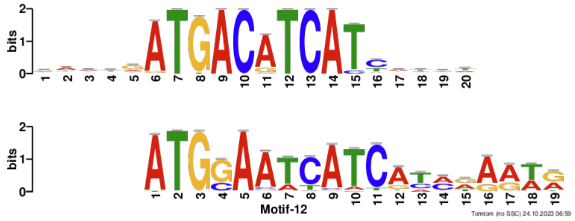

ZN214 (*H. sapiens*)
2.63e-06

ZN214_HUMAN.H11MO.0.C

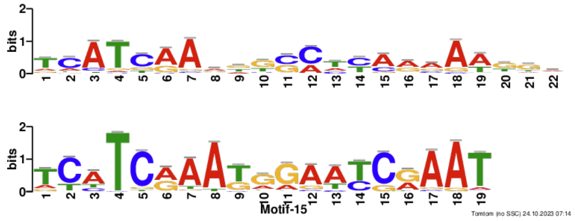

ZN582 (*H. sapiens*)
4.49e-09

ZN582_HUMAN.H11MO.0.C

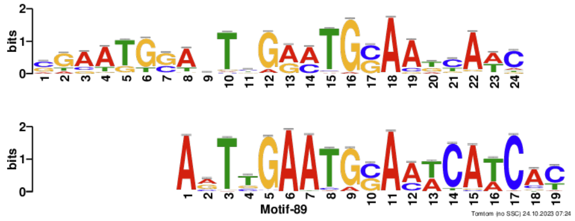

PO3F2 (*H. sapiens*)
1.05e-07

PO3F2_HUMAN.H11MO.0.A

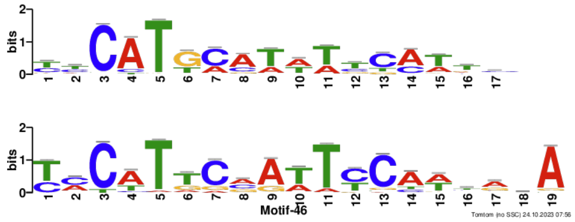

STA5B (*H. sapiens*)
1.05e-04

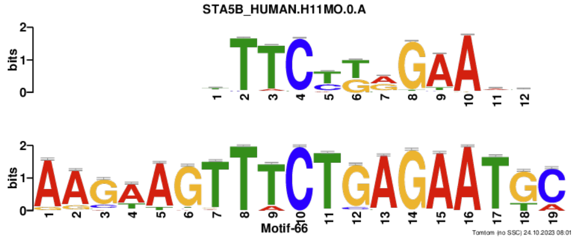

FOXM1 (*H. sapiens*)
5.40e-05

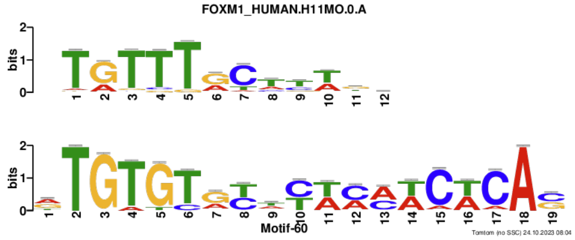

Z354A (*H. sapiens*)
1.89e-05

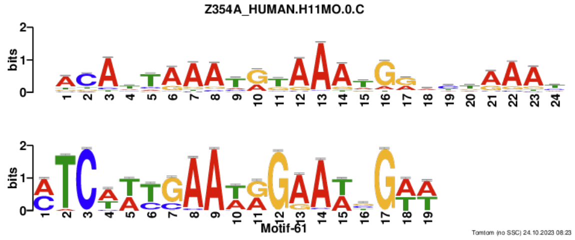

STA5A (*H. sapiens*)
1.75e-05

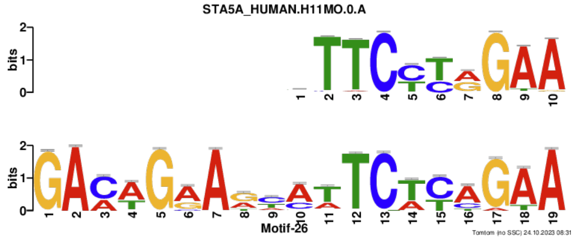

ZN667 (*H. sapiens*)
4.14e-05

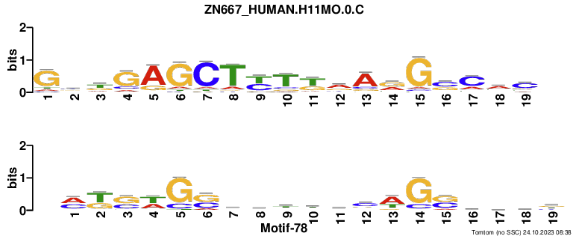

STAT4 (*H. sapiens*)
2.00e-05

PATZ1 (*H. sapiens*)
1.24e-09

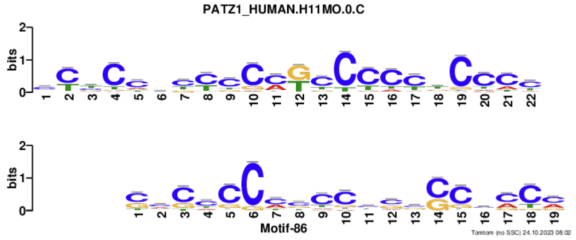

STAT6 (*H. sapiens*)
8.22e-05

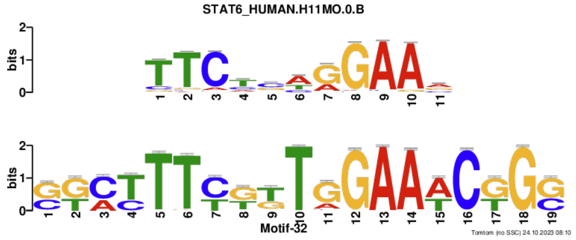

IRF3 (*H. sapiens*)
5.73e-05

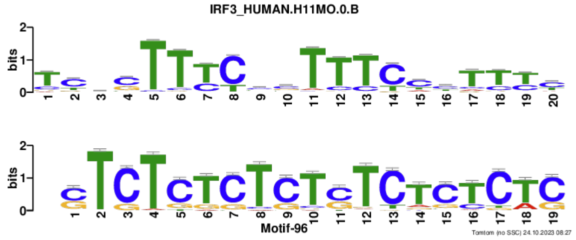

ZN563 (*H. sapiens*)
1.18e-06

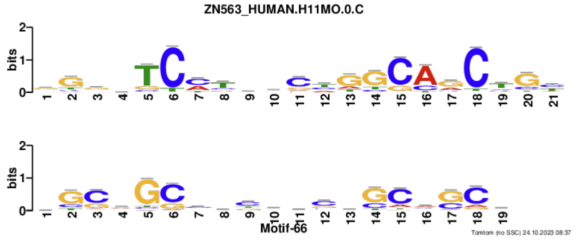

MNX1 (*H. sapiens*)
5.26e-05

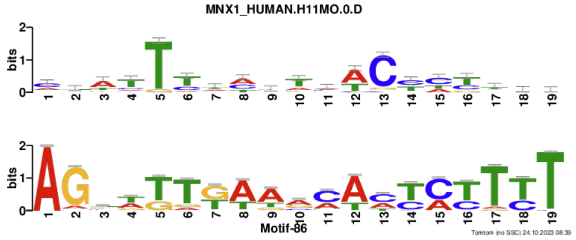

SP2 (*H. sapiens*)
4.36e-07

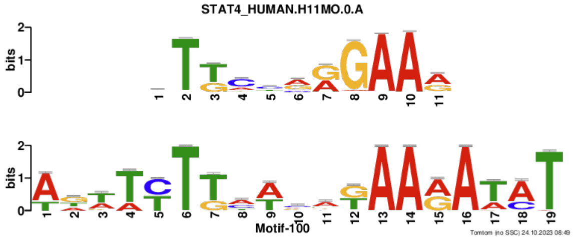

SP3 (*H. sapiens*)
7.25e-07

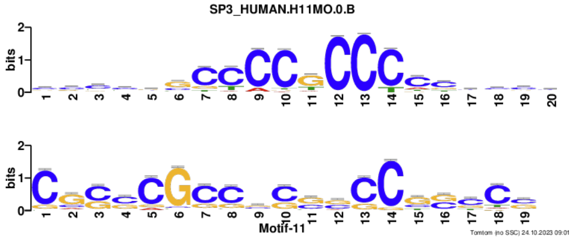

CPEB1 (*H. sapiens*)
1.43e-06

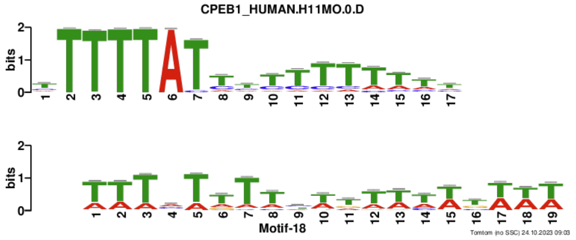

MAZ (*H. sapiens*)
4.14e-07

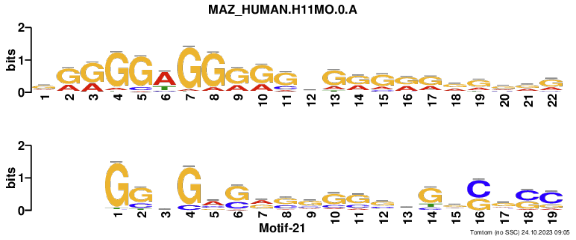

PO3F3 (*H. sapiens*)
4.67e-07

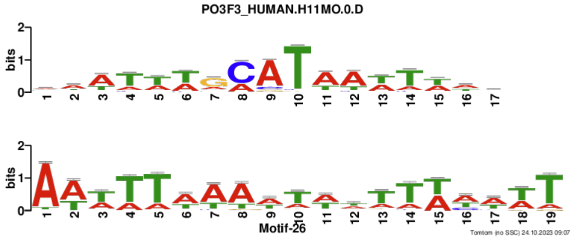

ZN335 (*H. sapiens*)
1.14e-06

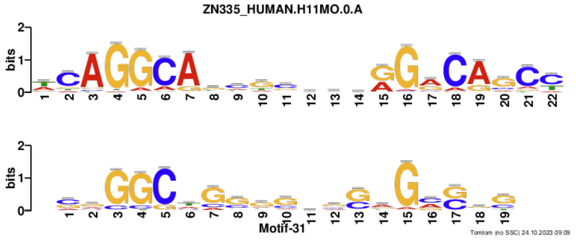

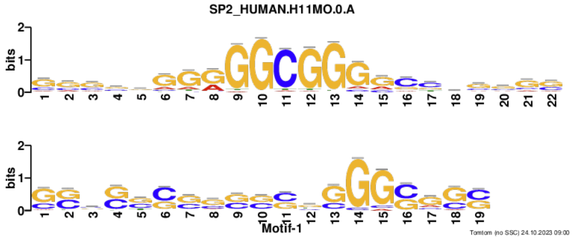

THAP1 (*H. sapiens*)
5.80e-06

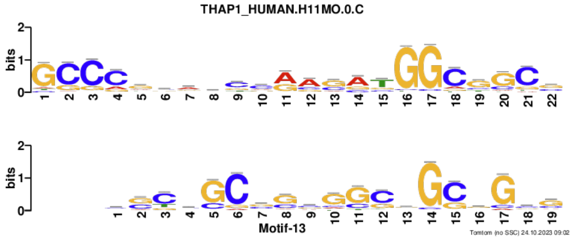

ZFX (*H. sapiens*)
2.44e-05

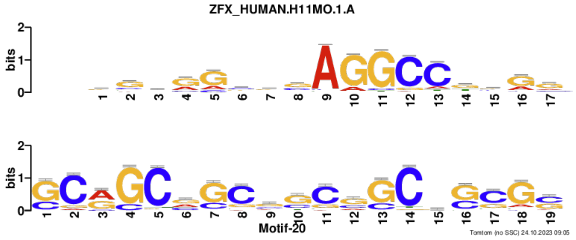

HXC10 (*H. sapiens*)
5.84e-06

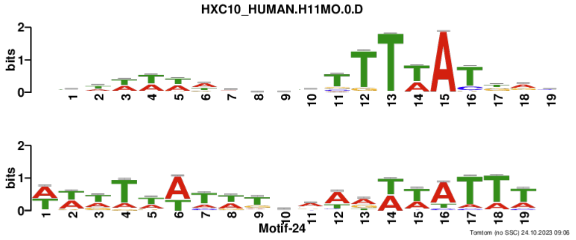

ZN148 (*H. sapiens*)
2.16e-08

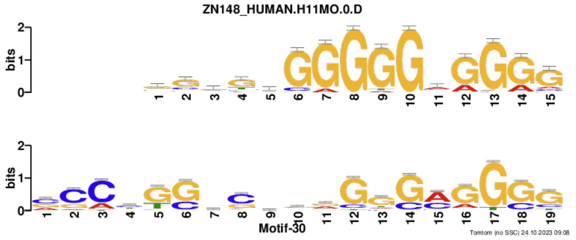

ANDR (*H. sapiens*)
6.36e-06

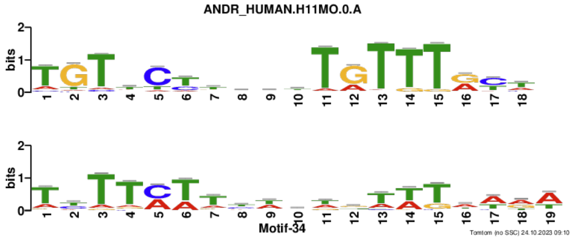

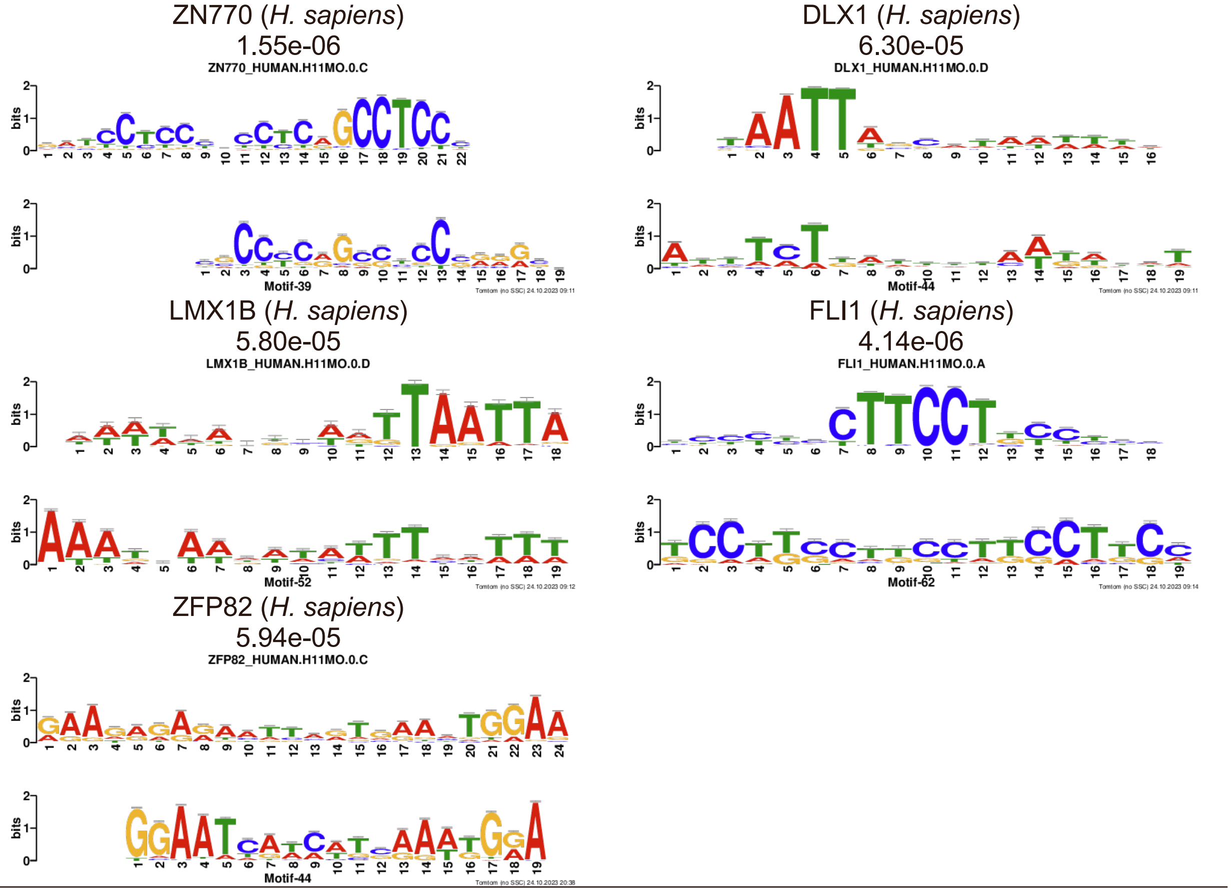

3. Note S3: Data acquisition

The raw sequencing data corresponding to GSE152144[2], in SRA format, were retrieved from the Sequencing Read Archive (SRA) using the accession code PRJNA638411[3]. These SRA files were then converted into FASTQ format utilizing the **fastq-dump** utility from the SRA Toolkit (v.3.0.2)[3]. For the alignment process, we employed Bowtie2 (v.2.4.1)[4] to accurately align the sequencing reads, now in FASTQ format, against the *H. sapiens* genome reference (hg19). This resulted in the generation of Sequence Alignment Map (SAM) files.

In the subsequent step, we converted these SAM files into more compressed Binary Alignment Map (BAM) files using SAMtools (v.1.16.1)[5] and performed a sorting procedure. Peak calling was conducted to identify narrow peaks, which were subsequently represented in Browser Extensible Data (BED) format. This was achieved by executing the **callpeak** function in MACS2 (v.2.2.9.1)[6].

For the benchmarking process, we required sequences to act as positive controls. Therefore, we employed the **getfasta** utility from BEDTools (version 2.29.1) to extract the sequences corresponding to these narrow peaks, resulting in their representation in FASTA format. These sequences served as the positive sequences for our benchmarking analysis.

For each positive sequence identified, we employed the R package Biostrings (v.2.66.0) to meticulously extract a random sequence of equivalent length from the *H. sapiens* genome, thereby generating a corresponding set of negative sequences. These positive and negative sequences were subsequently amalgamated to formulate the input dataset essential for rigorous benchmarking procedures.

Analytical tools such as Homer[7] and STREME[8] necessitate an auxiliary set of sequences to serve as a background reference. To fulfill this requirement, we again harnessed the capabilities of Biostrings, enabling

us to randomly curate a collection of sequences from the *H. sapiens* genome. Each sequence within this compilation mirrored the lengths of those found in our input dataset, ensuring consistency in our comparative analysis for benchmarking.

Furthermore, proChIPdb[9] conveniently furnishes us with narrow peaks, delineated in BED format, for each pertinent dataset. This provision allows us to directly incorporate these datasets into our benchmarking framework, streamlining the analysis process.

4. Note S4: Data preparation

We present TESA, a novel composite file format designed to integrate DNA sequences with their associated sequencing coverages. Constructing the TESA format necessitates a reference genome in FASTA format, narrow peaks in BED format, and sequencing coverage files in bigWig format. Initially, narrow peaks are alphabetically organized and uniformly adjusted to a standard length (typically 100 bp) using the **sort** and **awk** Shell utilities. Thereafter, the **bigWigToBedGraph** function[10] transitions the sequencing coverage files to the bedGraph format, enhancing versatility. Utilizing BEDTools, the **complement** function then standardizes sequencing coverages for the reverse complementary sequences of the narrow peaks. Conclusively, **getfasta** transforms narrow peaks from BED to FASTA format. These sequences, combined with bidirectional sequencing coverages, constitute the TESA file format.

5. Note S5: Calculation of weights in two-stage alignment

TESA employs a two-stage alignment strategy utilizing a matrix, M^h, of dimensions $2m \times n$. In M^h, the rows depict both the input sequences (odd rows) and their reverse complements (even rows). The columns mark the starting positions of segments, each of length l, on the sequences. Matrix entries encapsulate the normalized sequencing coverages. For segments s_{ij} from sequence s_i and s_{pq} from sequence s_p with k identical positions, TESA leverages the binomial distribution, denoted in **Equation 1**, to assess the statistical significance of their sequence similarity,

$$f(s_{ij}, s_{pq}) = -\lg\left(\sum_{k}^{l} B(l, k, 0.25)\right). \qquad \text{(Equation 1)}$$

If $B(l, k, 0.25) > 0.01$, TESA sets $f(s_{ij}, s_{pq}) = 0$. Then, TESA calculates the weight of the segment pair s_{ij} and s_{pq} by incorporating the sequencing coverage of both segments, as described by

$$f'(s_{ij}, s_{pq}) = f(s_{ij}, s_{pq}) \times (M_{ij}^h + M_{pq}^h). \qquad \text{(Equation 2)}$$

If $f(s_{ij}, s_{pq})$ surpasses a preset threshold (defaulted to 3) or if $f'(s_{ij}, s_{pq})$ is among the top α alignments (defaulted to 5) for all l-segment pairings, TESA augments both M_{ij}^1 and M_{pq}^1 by β. In TESA, the value of β is ascertained by evaluating the antecedent dinucleotides of s_{ij} and s_{pq}. When both segments share an identical preceding dinucleotide, β is set to 0.5; otherwise, it assumes a value of 1.0.

REFERENCES

1. Kulakovskiy, I.V., Vorontsov, I.E., Yevshin, I.S., Soboleva, A.V., Kasianov, A.S., Ashoor, H., Ba-alawi, W., Bajic, V.B., Medvedeva, Y.A., Kolpakov, F.A., and Makeev, V.J. (2015). HOCOMOCO: expansion and enhancement of the collection of transcription factor binding sites models. *Nucleic Acids Res 44*, D116-D125. 10.1093/nar/gkv1249.
2. Lai, W.K.M., Mariani, L., Rothschild, G., Smith, E.R., Venters, B.J., Blanda, T.R., Kuntala, P.K., Bocklund, K., Mairose, J., Dweikat, S.N., et al. (2021). A ChIP-exo screen of 887 Protein Capture Reagents Program transcription factor antibodies in human cells. *Genome Res 31*, 1663-1679. 10.1101/gr.275472.121.
3. Katz, K., Shutov, O., Lapoint, R., Kimelman, M., Brister, J R., and O'Sullivan, C. (2021). The Sequence Read Archive: a decade more of explosive growth. *Nucleic Acids Res 50*, D387-D390. 10.1093/nar/gkab1053.
4. Langmead, B., and Salzberg, S.L. (2012). Fast gapped-read alignment with Bowtie 2. *Nat Methods 9*, 357-359. 10.1038/nmeth.1923.

5. Danecek, P., Bonfield, J.K., Liddle, J., Marshall, J., Ohan, V., Pollard, M.O., Whitwham, A., Keane, T., McCarthy, S.A., Davies, R.M., and Li, H. (2021). Twelve years of SAMtools and BCFtools. *GigaScience 10*. 10.1093/gigascience/giab008.
6. Zhang, Y., Liu, T., Meyer, C.A., Eeckhoute, J., Johnson, D.S., Bernstein, B.E., Nusbaum, C., Myers, R.M., Brown, M., Li, W., and Liu, X.S. (2008). Model-based Analysis of ChIP-Seq (MACS). *Genome Biol 9*, R137. 10.1186/gb-2008-9-9-r137.
7. Duttke, S.H., Chang, M.W., Heinz, S., and Benner, C. (2019). Identification and dynamic quantification of regulatory elements using total RNA. *Genome Res 29*, 1836-1846. 10.1101/gr.253492.119.
8. Bailey, T.L. (2021). STREME: accurate and versatile sequence motif discovery. *Bioinformatics 37*, 2834-2840. 10.1093/bioinformatics/btab203.
9. Decker, K.T., Gao, Y., Rychel, K., Al Bulushi, T., Chauhan, Siddharth M., Kim, D., Cho, B.-K., and Palsson, Bernhard O. (2021). proChIPdb: a chromatin immunoprecipitation database for prokaryotic organisms. *Nucleic Acids Res 50*, D1077-D1084. 10.1093/nar/gkab1043.
10. Kent, W.J., Sugnet, C.W., Furey, T.S., Roskin, K.M., Pringle, T.H., Zahler, A.M., and Haussler, D. (2002). The human genome browser at UCSC. *Genome Res 12*, 996-1006. 10.1101/gr.229102.
