# Supplementary material for: A weighted two-stage sequence alignment framework to identify motifs from ChIP-exo data
Source: Patterns (N Y). 2024 Feb 2;5(3):100927. doi: 10.1016/j.patter.2024.100927 (PMC10935504; doi:10.1016/j.patter.2024.100927)
Supplement: Document S2. Article plus supplemental information [file mmc2.pdf]

Article

Patterns

A weighted two-stage sequence alignment framework to identify motifs from ChIP-exo data

Highlights

- TESA employs ChIP-exo for enhanced motif discovery through positional weighting
- TESA utilizes binomial and "bookend" models for precise motif length computation
- TESA excels in motif discovery across prokaryote/eukaryote data

Authors

Yang Li, Yizhong Wang, Cankun Wang, Anjun Ma, Qin Ma, Bingqiang Liu

Correspondence

qin.ma@osumc.edu (Q.M.),
bingqiang@sdu.edu.cn (B.L.)

In brief

TESA, a specialized ChIP-exo motif discovery framework, is developed, leveraging sequencing coverage with a positional weighting scheme. Incorporating a graph model and binomial distribution, alongside a novel "bookend" model, TESA enhances motif site prediction. Its efficacy is demonstrated through rigorous benchmarking studies on both prokaryotic and eukaryotic samples, highlighting its superior performance and marking a significant advancement in the field of genomic analysis.

Li et al., 2024, Patterns 5, 100927
March 8, 2024 © 2024 The Author(s).
https://doi.org/10.1016/j.patter.2024.100927

Patterns

CellPress
OPEN ACCESS

Article

A weighted two-stage sequence alignment framework to identify motifs from ChIP-exo data

Yang Li,[1,4] Yizhong Wang,[2,4] Cankun Wang,[1] Anjun Ma,[1] Qin Ma,[1,3,*] and Bingqiang Liu[2,5,*]
[1]Department of Biomedical Informatics, College of Medicine, The Ohio State University, Columbus, OH 43210, USA
[2]School of Mathematics, Shandong University, Jinan, Shandong 250100, China
[3]Pelotonia Institute for Immuno-Oncology, The James Comprehensive Cancer Center, The Ohio State University, Columbus, OH 43210, USA
[4]These authors contributed equally
[5]Lead contact
*Correspondence: qin.ma@osumc.edu (Q.M.), bingqiang@sdu.edu.cn (B.L.)
https://doi.org/10.1016/j.patter.2024.100927

THE BIGGER PICTURE Due to the significant role of transcription factors (TFs) in gene regulation, deciphering their binding patterns is key to understanding critical genomic regulatory elements. The ChIP-exonuclease (ChIP-exo) technology, known for its higher resolution and reduced noise, has advanced research in this area. This paper introduces an advanced motif discovery algorithm that leverages the refined resolution of ChIP-exo by integrating a positional weighting scheme and a "bookend" model. Its adaptability for future comprehensive studies that integrate TF binding with gene expression analysis makes the algorithm a promising tool for deducing gene regulatory networks.

SUMMARY

In this study, we introduce TESA (weighted two-stage alignment), an innovative motif prediction tool that refines the identification of DNA-binding protein motifs, essential for deciphering transcriptional regulatory mechanisms. Unlike traditional algorithms that rely solely on sequence data, TESA integrates the high-resolution chromatin immunoprecipitation (ChIP) signal, specifically from ChIP-exonuclease (ChIP-exo), by assigning weights to sequence positions, thereby enhancing motif discovery. TESA employs a nuanced approach combining a binomial distribution model with a graph model, further supported by a "bookend" model, to improve the accuracy of predicting motifs of varying lengths. Our evaluation, utilizing an extensive compilation of 90 prokaryotic ChIP-exo datasets from proChIPdb and 167 *H. sapiens* datasets, compared TESA's performance against seven established tools. The results indicate TESA's improved precision in motif identification, suggesting its valuable contribution to the field of genomic research.

INTRODUCTION

Sequence-binding proteins, pivotal in orchestrating gene regulation, engage specific DNA or RNA sequences, thereby exerting a profound influence through transcriptional modulation, chromosomal structural dynamics, and epigenetic governance, encapsulating entities like transcription factors (TFs), RNA-binding proteins, and chromatin-associated proteins.[1–9] Understanding TF-binding patterns (motifs) elucidates key genomic regulatory elements like enhancers and promoters, illuminating the intricate interaction networks among TFs and proteins involved in transcriptional regulation.[10–12] In this work, we present TESA (weighted two-stage alignment), an advanced motif discovery algorithm (Figure 1), and, through a meticulous and comprehensive evaluation using chromatin immunoprecipitation-exonuclease (ChIP-exo) data,[13] demonstrate its advantages over mainstream algorithms.

In contrast to established motif discovery algorithms such as BoBro,[14] Homer,[15] MEME,[16] MFMD,[17] PEnGmotif,[18] STREME,[9] and XXmotif,[19] TESA distinctly leverages the augmented resolution offered by ChIP-exo technology, a facet that markedly enhances its motif discovery precision relative to ChIP sequencing (ChIP-seq) methodologies.[20] While TESA employs the two-stage alignment procedure characteristic of BoBro,[14] it innovatively amplifies the identification of potential motif instances by judiciously integrating positional sequencing coverage into the weighting scheme during the alignment process. This nuanced integration allows TESA to weight potential motif instances, thereby refining alignment specificity and improving the predictive accuracy of motif instances. In contrast to TESA, the majority of existing algorithms deploy methodologies that either hypothesize that TFs exhibit a preferential binding affinity toward peak centers, as exemplified by MEME,[16] or uniformly treat all segments with equal significance,

This is an open access article under the CC BY license (http://creativecommons.org/licenses/by/4.0/).

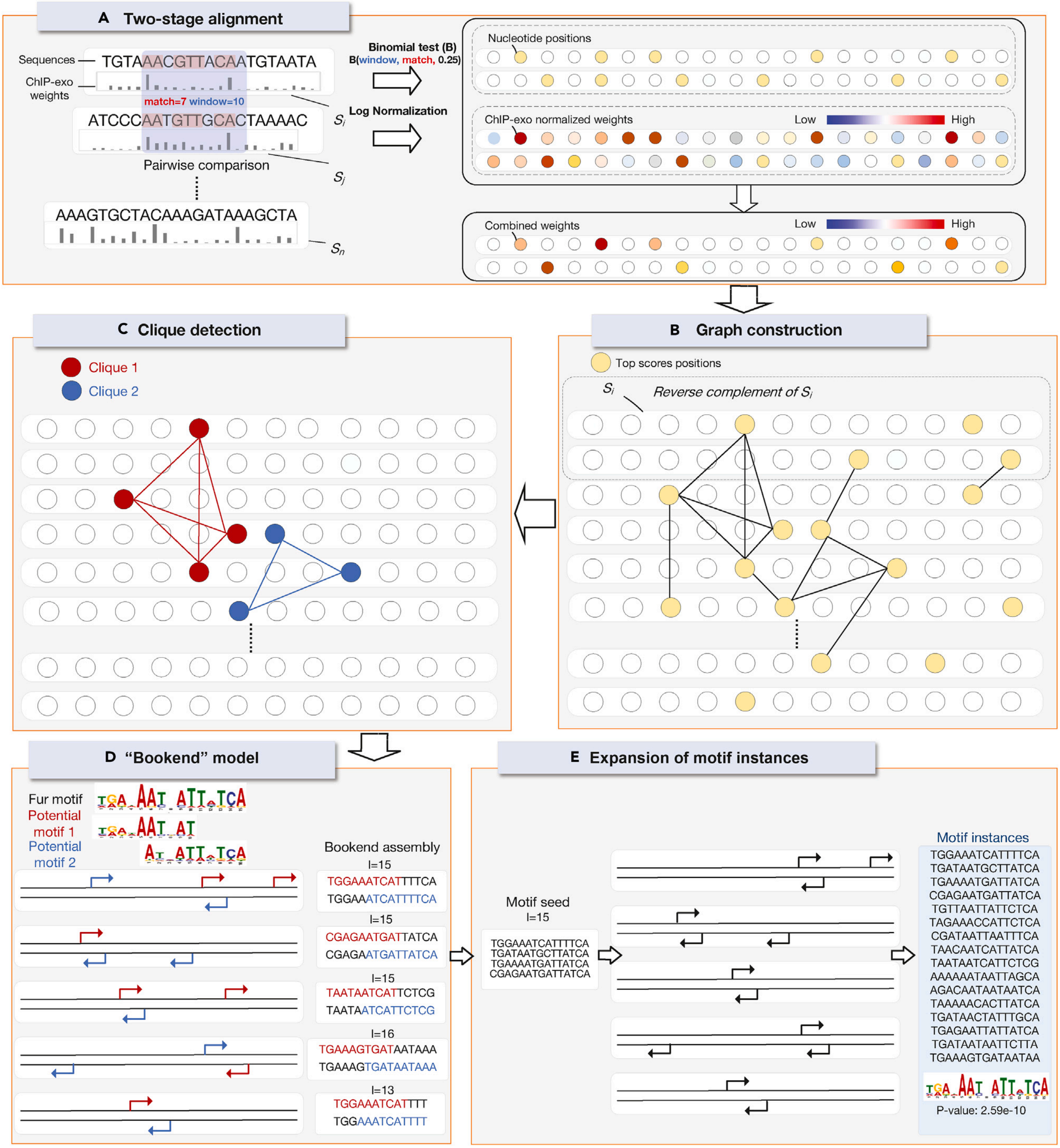

Figure 1. Overview of the TESA motif detection framework

(A) Sequences undergo a two-stage alignment. During this process, segments from distinct sequences are compared, with their statistical significance being evaluated through a binomial distribution. Subsequent log normalization then assigns a combined weight for each sequence position.

(B) The top-scoring positions from the two-stage alignment are chosen as vertices to construct a graph, incorporating both input sequences as well as their reverse complements.

(C) Within the constructed graph, cliques are detected utilizing a heuristic algorithm. These cliques serve as potential motifs.

(D) A specialized "bookend" method is deployed to optimize the length of the motifs. This optimization assesses the sequential overlaps between pairs of potential motifs. Once assembled using the bookend method, all motif instances act as a seed for the ensuing step.

(E) Concluding the process, motif instances undergo expansion by selecting segments with elevated motif match scores. Based on these instances, the motif position weight matrix (PWM) is structured.

as demonstrated by algorithms like BoBro,[14] XXmotif,[19] and STREME,[9] among others.

TESA refines motif widths utilizing a unique "bookend" model, underpinned by a binomial distribution test, starkly contrasting approaches such as those adopted by BoBro,[14] Homer,[15] MEME,[16] and STREME,[9] which methodically explore all motif widths within a range predetermined by the user. XXmotif fine-tunes motif widths through an iterative process, which encompasses extension or reduction of up to two positions at both ends, subsequently evaluating the refined motifs utilizing *E*-values.[19] However, the strategy of bidirectional extension hinges upon the statistical derivation of *E*-values, potentially subverting biological relevance and generating a surfeit of redundant motifs. In contrast, TESA implements a bookend model, rigorously examining the sequential proximity of sites between each pair of potential motifs, thereby offering a thorough and direct assessment of their coincident co-occurrence. Based on the statistical significance of the co-occurrence, TESA elects to merge two potential motifs to form a longer one or to treat them as distinct entities.

Through the integration of a positional weighting scheme and a bookend model, TESA facilitates robust and precise motif discovery, capitalizing on the unique benefits offered by ChIP-exo over ChIP-seq.[13] TESA allocates weights to sequence positions in accordance with the positional sequencing coverage inherent to ChIP-exo, thereby delineating precise binding affinities. Given the ability of ChIP-exo to pinpoint narrower enriched regions, the bookend model adeptly discerns subtle variations and positional nuances in TF binding that might be obscured within the broader peaks typical of ChIP-seq. Furthermore, the accurate localization of TF-DNA interaction sites by ChIP-exo mitigates background noise, attenuating the influence of spurious or non-specific binding events during the implementation of the bookend model. Crucially, ChIP-exo's precision enables TESA to distinctly assess closely located or overlapping binding occurrences. In essence, TESA exploits the high resolution, diminished noise, and precise localization of ChIP-exo data to augment motif discovery.

In the following sections of this article, we delve into the intricacies of the TESA algorithm and showcase experimental results that benchmark its performance against seven prevalent motif discovery algorithms. Our experimental comparisons span motif discovery across ChIP-exo datasets from both prokaryotic and eukaryotic organisms. In each instance, we evaluate performance by discerning ChIP-exo peaks (positive sequences) and DNA sequences (negative sequences), the latter being randomly selected from entire genomes. Moreover, we validate the motifs predicted by employing Tomtom[21] to gauge their similarity to motifs documented in collectTF (for prokaryotes)[22] and HOCOMOCO v.11 (for eukaryotes),[23] respectively.

RESULTS

TESA excels in distinguishing TF-binding sequences

The efficacy of a motif discovery algorithm, when deployed on a TF ChIP-exo dataset, hinges on its proficiency in discriminating between DNA sequences that are bound by the ChIP-enriched TF and those that are not. The precision of these algorithms is assessed through a comparative analysis of their binary classification performance between these two distinct categories of DNA sequences. This evaluation employs the metric of partial area under the receiver operating characteristic curve (pAUC),[24] with an emphasis on both specificity and sensitivity. The assessment parameters are set to measure the true positive rate and false positive rate within the intervals of $(0.8, 1.0)$ and $(0.0, 0.2)$, respectively, thereby focusing on the algorithm's performance in critical regions of the classification threshold. Each algorithm is executed under its standard configuration. Specifically, for algorithms that establish a background model based on their input sequences—namely, Homer, PEnGmotif, and STREME—we ensure methodological consistency by mandating the selection of an equivalent number of randomly chosen DNA sequences that mirror the lengths of the input sequences. Implementation parameters for all benchmarked algorithms are detailed in the Note S1.

Figures 2A and 2B demonstrate that TESA-cov, TESA with sequence coverage integration, achieves pAUC values matching or surpassing those of competing algorithms in our study, which analyzed 90 proChIPdb datasets of 85 TFs. TESA-cov showcases notable median pAUCs of 0.97 for sensitivity (pAUC [sensitivity]) and 0.94 for specificity (pAUC [specificity]), as highlighted in Figures 2A and 2B. TESA's variant that processes only DNA sequences, termed TESA-basic, along with BoBro and MFMD, mirrors TESA-cov's performance, each registering a median pAUC (sensitivity) of 0.97 and a median pAUC (specificity) of 0.94. Meanwhile, other algorithms in our assessment, including XXmotif, MEME, and Homer, manifest a significant decline in both pAUC metrics, falling below 0.80. Notably, XXmotif's pAUC (specificity) is particularly diminished, scoring less than 0.60.

In the analysis of the 167 *H. sapiens* datasets of 67 TFs, as shown in Figures 2C and 2D, TESA-cov and TESA-basic consistently outperformed other evaluated methodologies. Specifically, TESA-cov registers a slight enhancement in pAUC (sensitivity), achieving a median value of 0.82, compared to TESA-basic's median of 0.81. BoBro's performance on these datasets lags, particularly when assessed by pAUC (sensitivity). This underperformance is evident when contrasted with other methods such as MEME, STREME, PEnGmotif, and Homer, as demonstrated in Figure 2C. However, in terms of pAUC (specificity), BoBro remains competitive, showing only a modest deviation from the values achieved by TESA-cov and TESA-basic. Distinctly different from its performance on proChIPdb datasets, XXmotif and MFMD trail the pack, with both pAUC (sensitivity) and pAUC (specificity) falling below 0.60.

TESA accurately recovers motif PWMs

The precision of motif discovery algorithms is assessed by quantifying the similarity between each discovered motif's position weight matrix (PWM) and the known motif corresponding to the ChIP-enriched TF. This quantification is executed utilizing the Tomtom motif comparison tool[21] against collectTF and HOCOMOCO v.11, respectively. Throughout the experiments conducted for this study, Tomtom is operated via its default command-line parameters. To ensure that elevated scores are indicative of more accurate motifs, we adopt the negative base-10 logarithm of the Tomtom p value, derived from the

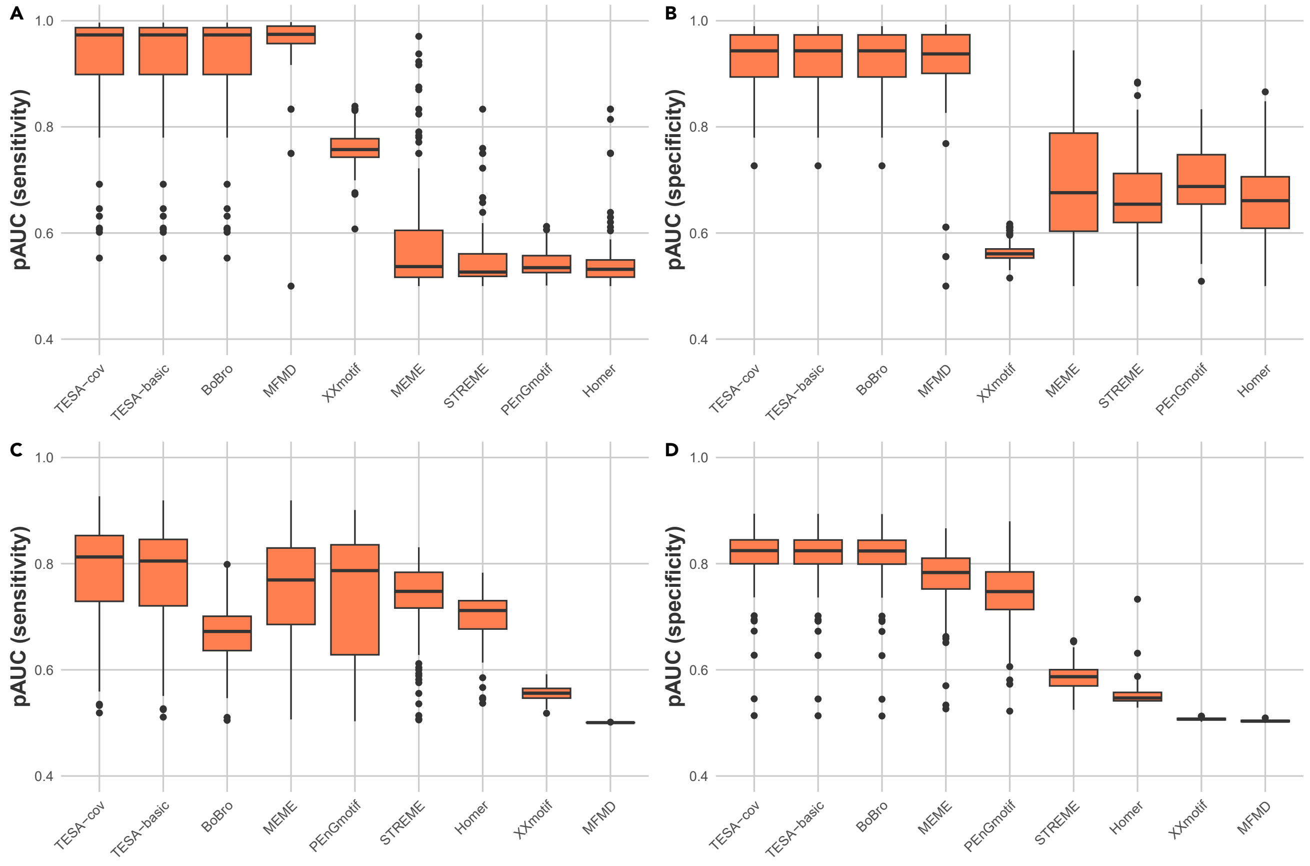

Figure 2. Comparative assessment of sequence bi-classification for TESA versus existing methods

(A) pAUC (sensitivity) performance across proChIPdb datasets.

(B) pAUC (specificity) metrics for the same proChIPdb datasets.

(C) Comparison of pAUC (sensitivity) on *H. sapiens* datasets.

(D) Corresponding pAUC (specificity) evaluations for the *H. sapiens* datasets.

similarity assessment between the identified motif and the reference motif, as our definitive motif similarity score.

In the assessment of the ability of TESA, alongside other parallel algorithms, to faithfully reconstruct PWMs for proChIPdb datasets, Figure 3A reveals that the PWMs discerned by both TESA-cov and TESA-basic typically demonstrate heightened congruence with the curated benchmark standards. This is in comparison to PWMs identified by other algorithms examined in this study. Specifically, TESA-cov and TESA-basic manifest median motif similarity scores of 16.61 and 15.47, respectively. Additionally, we examine the ranks of motifs identified that align with the motifs of ChIP-enriched TFs that lead to the most statistically significant p values. In this context, TESA-cov and TESA-basic consistently register superior average ranks of 5.25 and 4.875, outpacing the majority of alternative methods, including Homer (10.90), MEME (8.00), PEnGmotif (15.68), and XXmotif (61.23). It is noteworthy that while BoBro and STREME achieve commendable average ranks, at 3.40 and 1.82, respectively, the motifs they identify exhibit diminished motif similarity scores. Regarding MFMD, given its propensity to yield either no motifs or a singular motif per dataset, it is excluded from our comparative analysis.

Utilizing a methodological approach analogous to that employed for the proChIPdb datasets, the 167 *H. sapiens* datasets were subjected to a comparable evaluation, as depicted in Figure 3B. Both TESA-cov and TESA-basic emerge with superior motif similarity scores, registering median values of 9.49 and 9.33, respectively, surpassing other methods. Notably, in contrast to the observations from proChIPdb datasets, none of the motifs identified by STREME, MFMD, and PEnGmotif align with the motifs of ChIP-enriched TFs. Furthermore, for motifs congruent with those of the ChIP-enriched TFs, those discerned by TESA-cov, TESA-basic, and BoBro exhibit marginally superior average ranks, registering at 6.29, 5.57, and 5.0, respectively. In comparison, MEME, Homer, and XXmotif attain average ranks of 6.42, 6.71, and 52.07, respectively. Note S2 shows sequence logos for the best TESA-cov motifs, and their Tomtom p values, aligned to the logo of the reference motifs.

DISCUSSION

This study's primary contributions are 2-fold: first, the introduction of the innovative TESA algorithm, and second, a thorough and systematic performance assessment of TESA in conjunction

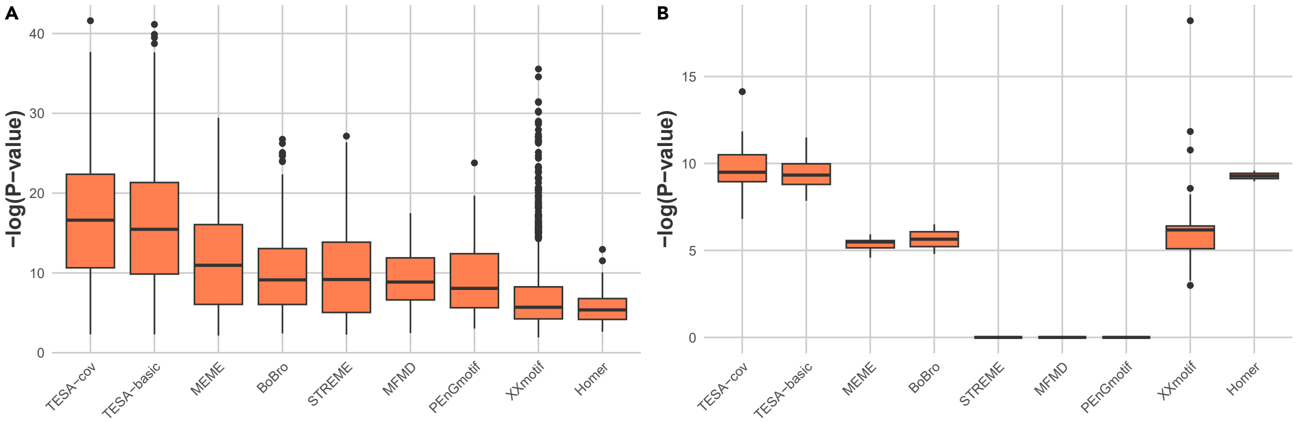

Figure 3. Comparative analysis of motif similarity scores between detected and reference motifs
(A) Assessment of motif similarity scores within proChIPdb datasets.
(B) Examination of motif similarity scores within *H. sapiens* datasets.

with seven other established motif discovery algorithms. The algorithms selected for this comparative analysis represent the most prevalently employed tools in the realm of ChIP-seq/-exo motif analysis, encompassing BoBro,[14] Homer,[15] MFMD,[17] MEME,[16] PEnGmotif,[18] STREME,[9] and XXmotif.[19] Our evaluative methodology is distinctive in its construction of datasets, which comprise ChIP-exo narrow peaks (classified as positive sequences) and sequences arbitrarily chosen from genomes (classified as negative sequences). This approach facilitates an astute evaluation of sequence bi-classification performance. An ancillary yet significant contribution of this research is the introduction of the bookend model within TESA, which presents a methodological innovation in the precise determination of motif widths. This model circumvents the need for the labor-intensive and computationally demanding process of exhaustively enumerating varying widths, thereby enhancing efficiency and practicality in motif discovery endeavors.

EXPERIMENTAL PROCEDURES

Resource availability

Lead contact

The lead contact for questions about this paper is Bingqiang Liu, who can be reached at bingqiang@sdu.edu.cn.

Materials availability

No unique materials were generated from this study.

Data and code availability

The datasets referenced in this study's experimental procedures are publicly accessible,[25,26] ensuring transparency and reproducibility in our research. No new data were generated in this work.

The source code for TESA, accompanied by an extensive tutorial, is accessible via the following GitHub repository (https://github.com/OSU-BMBL/tesa) and Zenodo (https://doi.org/10.5281/zenodo.8056408).[27]

Additional data analysis details used in this paper are available upon request from the lead contact, ensuring comprehensive data transparency and facilitating further research exploration.

Methodology

Data acquisition

To assess the performance of motif discovery algorithms utilizing ChIP-exo data, we identified 90 (all available) and 167 TF ChIP-exo experiments in prokaryotes (sourced from proChIPdb[25]) and *H. sapiens* (derived from GEO: GSE152144 [26]), respectively. Comprehensive details regarding the preprocessing of ChIP-exo datasets and the generation of negative sequences for each dataset can be found in Note S3.

Data preparation

First, an input sequence dataset is meticulously prepared, which encompasses a reference genome file (in FASTA format), a narrow peak file (in BED format), and one or multiple sequencing coverage file(s) (in BigWig format). Subsequently, the narrow peaks, initially represented in BED format, are converted into FASTA format, employing BEDTools as a facilitative utility.[28] Following this transformation, sequencing coverages are assigned to each position within the narrow peaks. Ultimately, the sequencing coverage of each position ($x_i, i = 1, \ldots, n$) is normalized as per Equation 1, ensuring that the sequencing coverages across diverse narrow peaks are bounded within the $[0, 1]$ interval. The narrow peaks, formatted in FASTA, in conjunction with the normalized sequencing coverages, are coalesced into a singular file, herein referred to as the TESA format, which subsequently serves as the input for TESA. In instances where users abstain from providing sequencing coverage file(s), TESA defaults to accepting the FASTA file as input. Exhaustive information pertaining to the methodologies employed for data preparation is elaborately delineated in Note S4.

$$x_i = \frac{\log(1+x_i)}{\max_{i=1,2,\ldots,n}(\log(1+x_i))}, i = 1, \cdots, n. \qquad \text{Equation 1}$$

Step 1: Two-stage alignment

Without loss of generality, it is assumed that the input consists of m sequences, each of equal length n, with each position assigned a normalized sequencing coverage (Figure 1A). Subsequently, TESA constructs a matrix M^h of dimensions $2m \times n$. The rows of M^h represent both the input sequences (odd rows) and their reverse complementary sequences (even rows), while columns indicate the starting positions of segments of length l on sequences. The entries of the matrix convey the normalized sequencing coverages.

TESA scores sequence positions through the development of a weighted, enhanced version of BoBro's two-stage alignment procedure.[14] In the initial alignment round, TESA allocates weights to each pair of segments, each of length l ($l = 14$ as default), between disparate sequences, predicated upon both string similarity and sequencing coverage. For segments s_{ij} from sequence s_i and s_{pq} from sequence s_p, which exhibit k identical positions, TESA employs the binomial distribution, as articulated in Equation 2, to evaluate the statistical significance of their string similarity,

$$f(s_{ij}, s_{pq}) = -\lg\left(\sum_{k}^{l} B(l, k, 0.25)\right). \qquad \text{Equation 2}$$

TESA computes the weight of the segment pair, s_{ij} and s_{pq}, by integrating the sequencing coverage of both segments, defined as

$$f'(s_{ij}, s_{pq}) = f(s_{ij}, s_{pq}) \times \left(M^h_{ij} + M^h_{pq}\right). \quad \text{Equation 3}$$

TESA constructs a matrix M^1 to delineate the weight of segments, utilizing metrics f and f' as follows. Initialized with zeros, M^1 is subsequently updated through exhaustive comparison of sequence pairs. Specifically, for each pair of sequences, s_i and s_p, increments of one are applied to M^1_{ij} and M^1_{pq} if $f(s_{ij}, s_{pq})$ exceeds a defined threshold (3 as default) or $f'(s_{ij}, s_{pq})$ ranks within the top α (defaulted to 5) across all the l-segment alignments. Detailed insights into the two-stage alignment utilized in this study are comprehensively presented within the Note S5.

Should s_{ij} and s_{pq} represent authentic motif instances, the apex value across all l-segment alignments between s_i and s_p is likely observed at proximal segments of s_{ij} and s_{pq}, respectively, within two base pairs.[14] Hence, to incorporate such observation, TESA recomputes $f'(s_{ij}, s_{pq})$ utilizing M^1. This is formalized as

$$f'(s_{ij}, s_{pq}) = f(s_{ij}, s_{pq}) \times \max_{\substack{j-2 \le j' \le j+2 \\ q-2 \le q' \le q+2}} \left(M^1_{ij'} + M^1_{pq'}\right). \quad \text{Equation 4}$$

Using the same approach as the derivation of M^1, TESA constructs M^2 based on the values of f and f'. Following this, both M^h and M^2 are normalized by dividing each row by its maximum value. Subsequently, TESA refines M^2 by adding the corresponding values from M^h to M^2. The updated M^2 matrix then serves as the input for step 2.

Step 2: Graph construction

TESA commences by initializing an empty graph, denoted as G. Subsequently, for each l-segment pair s_{ij} and s_{pq}, the function $f'(s_{ij}, s_{pq})$ is computed based on M^2 as

$$f'(s_{ij}, s_{pq}) = f(s_{ij}, s_{pq}) \times \left(M^2_{ij} + M^2_{pq}\right). \quad \text{Equation 5}$$

If $f'(s_{ij}, s_{pq})$ ranks among the top β (where the default $\beta = 3$) for all l-segments pairs between sequences s_i and s_p, then s_{ij} and s_{pq} are added to G as vertices and are interconnected by an edge. Analogous to the approach adopted by BoBro,[14] the two-stage alignment in step 1 augments the signal-to-noise ratio of the resultant graph G (Figure 1B). Subsequently, this graph G is utilized as the input for step 3.

Step 3: Clique detection

TESA identifies all disjoint maximal cliques in G, denoted as potential motifs, employing the methodology consistent with BoBro (Figure 1C).[14] Should the user specify a range for the variable l, TESA iteratively executes steps 1–3, facilitating the recognition of potential motifs with varying lengths.[14] The identified potential motifs subsequently serve as the input for step 4.

Step 4: Optimization of motif width using the bookend model

For each pair of potential motifs c_i and c_j, encompassing n_i and n_j instances, respectively, TESA computes o_{ij}, denoting the count of overlapped instance pairs where each instance is derived from c_i and c_j, respectively, and simultaneously resides within a window of width d (default: 25 bp) (Figure 1D). In the computation of o_{ij}, reverse complementary sequences are additionally taken into account. Let us assume $n_i \ge n_j$ without loss of generality. The significance of o_{ij} is approximated by TESA using the equation

$$P(o_{ij}) = \sum_{k=o_{ij}}^{n_j} \binom{n_j}{k} p^k (1-p)^{n_j-k}. \quad \text{Equation 6}$$

Here, $p = (dn_i)/(mn)$ represents the estimated probability that a pair of randomly selected instances from c_i and c_j are within distance d. If o_{ij} is deemed statistically significant ($P(o_{ij}) < 0.05$), TESA integrates potential motifs c_i and c_j while maintaining their intersecting instances. Given that instances from c_i and c_j might overlap with varying numbers of nucleotides, TESA retains instances corresponding to the modal number of overlapping nucleotides, denoted as l_o. The curated instances constitute a motif, and this collection of motifs serves as the input for step 5.

Step 5: Expansion of motif instances

For every motif, denoted as x, identified in step 4 with a designated length $l_x = 2l - l_o$, TESA constructs its PWM (Figure 1E). This is mathematically represented by

$$PWM_x = \left(\log \frac{p(i,j)}{q(i)}\right)_{4 \times l_x}. \quad \text{Equation 7}$$

Here, (i,j) represents the probability that nucleotide type i occurs at position j within the motif. Conversely, $q(i)$ indicates the background probability of nucleotide type i being present in the entire genome. The motif match score for any given sequence segment $s_{i'j'}$ of length l_x based on PWM_x is computed by TESA as follows:

$$S(s_{i'j'}, x) = \frac{1}{2}\left(\max_{j'-t \le j'' \le j'} M^h_{i'j''} + \max_{j' \le j'' \le j'+l_o+t} M^h_{i'j''}\right)\left(\sum_{i=1}^{4}\sum_{j=1}^{l_x} PWM_x(i,j) \cdot I(i,j)\right). \quad \text{Equation 8}$$

In this equation, $I(i,j)$ signifies whether the j-th nucleotide of $s_{i'j'}$ corresponds to nucleotide type i. Furthermore, M^h represents the scoring metric, as delineated in step 1. The term t stands as a modifiable parameter and possesses a default value of 2. Utilizing Equation 8, TESA evaluates the motif match scores across all instances of motif x in addition to all the l_x segments of the input sequences. Subsequently, TESA refines motif x by assimilating those l_x segments from the input sequences whose motif match scores, when compared to PWM_x, exceed those of at least one instance of motif x. Adhering to this methodology, TESA expands the sets of motif instances, upon which the PWMs are subsequently reconstructed.

SUPPLEMENTAL INFORMATION

Supplemental information can be found online at https://doi.org/10.1016/j.patter.2024.100927.

ACKNOWLEDGMENTS

The authors express their gratitude to Dr. Shuangquan Zhang for his critical evaluation of the motif-finding programs. Furthermore, the manuscript significantly benefits from Dr. Jing Jiang's and Dr. Zhaoqian Liu's expert contributions in methodology refinement and figure design. The authors also extend their gratitude to Dr. Qi Wang, Di Li, and Qiuqin Wu for their meticulous assistance in software testing and their constructive feedback on the project. This research received financial support from the National Key R&D Program of China (grant no. 2020YFA0712400), the National Natural Science Foundation of China (NSFC; grant nos. 62272270 and 11931008), and the Shandong University Multidisciplinary Research and Innovation Team of Young Scholars (grant no. 2020QNQT017).

AUTHOR CONTRIBUTIONS

Q.M. and B.L. conceptualized the foundational hypothesis and orchestrated the comprehensive analytical framework. Y.L. executed the experimental procedures and finalized the manuscript, receiving critical assistance from Y.W. C.W. was responsible for the development of the primary software infrastructure. A.M. provided instrumental insights and recommendations pertaining to the data analysis strategies.

DECLARATION OF INTERESTS

The authors declare no competing interests.

Received: June 14, 2023
Revised: August 18, 2023
Accepted: January 10, 2024
Published: February 2, 2024

CellPress
OPEN ACCESS

REFERENCES

1. Li, Y., Ni, P., Zhang, S., Li, G., and Su, Z. (2019). ProSampler: an ultrafast and accurate motif finder in large ChIP-seq datasets for combinatory motif discovery. Bioinformatics *35*, 4632–4639.
2. Zhang, S., Ma, A., Zhao, J., Xu, D., Ma, Q., and Wang, Y. (2022). Assessing deep learning methods in cis-regulatory motif finding based on genomic sequencing data. Briefings Bioinf. *23*, bbab374.
3. Liu, B., Yang, J., Li, Y., McDermaid, A., and Ma, Q. (2018). An algorithmic perspective of de novo cis-regulatory motif finding based on ChIP-seq data. Briefings Bioinf. *19*, 1069–1081.
4. Ni, P., and Su, Z. (2019). Deciphering epigenomic code for cell differentiation using deep learning. BMC Genom. *20*, 709.
5. Ma, H., Wen, H., Xue, Z., Li, G., and Zhang, Z. (2022). RNANetMotif: Identifying sequence-structure RNA network motifs in RNA-protein binding sites. PLoS Comput. Biol. *18*, e1010293.
6. Niu, M., Tabari, E., Ni, P., and Su, Z. (2018). Towards a map of cis-regulatory sequences in the human genome. Nucleic Acids Res. *46*, 5395–5409.
7. Yang, J., Ma, A., Hoppe, A.D., Wang, C., Li, Y., Zhang, C., Wang, Y., Liu, B., and Ma, Q. (2019). Prediction of regulatory motifs from human Chip-sequencing data using a deep learning framework. Nucleic Acids Res. *47*, 7809–7824.
8. Wang, Y., Li, Y., Wang, C., Lio, C.W.J., Ma, Q., and Liu, B. (2024). CEMIG: Prediction of the cis-regulatory motif using the de Bruijn graph from ATAC-seq Briefings Bioinf. *25*, bbad505.
9. Bailey, T.L. (2021). STREME: accurate and versatile sequence motif discovery. Bioinformatics *37*, 2834–2840.
10. Li, Y., Ma, A., Wang, Y., Wang, C., Chen, S., Fu, H., Liu, B., and Ma, Q. (2022). Enhancer-driven gene regulatory networks inference from single-cell RNA-seq and ATAC-seq data. Preprint at bioRxiv. https://doi.org/10.1101/2022.12.15.520582.
11. Ma, A., Wang, X., Li, J., Wang, C., Xiao, T., Liu, Y., Cheng, H., Wang, J., Li, Y., Chang, Y., et al. (2023). Single-cell biological network inference using a heterogeneous graph transformer. Nat. Commun. *14*, 964.
12. Li, Y., Ma, A., Mathé, E.A., Li, L., Liu, B., and Ma, Q. (2020). Elucidation of biological networks across complex diseases using single-cell omics. Trends Genet. *36*, 951–966.
13. Rhee, H.S., and Pugh, B.F. (2011). Comprehensive genome-wide protein-DNA interactions detected at single-nucleotide resolution. Cell *147*, 1408–1419.
14. Li, G., Liu, B., Ma, Q., and Xu, Y. (2011). A new framework for identifying cis-regulatory motifs in prokaryotes. Nucleic Acids Res. *39*, e42.
15. Duttke, S.H., Chang, M.W., Heinz, S., and Benner, C. (2019). Identification and dynamic quantification of regulatory elements using total RNA. Genome Res. *29*, 1836–1846.
16. Bailey, T.L., Williams, N., Misleh, C., and Li, W.W. (2006). MEME: discovering and analyzing DNA and protein sequence motifs. Nucleic Acids Res. *34*, W369–W373.
17. Caldonazzo Garbelini, J.M., Kashiwabara, A.Y., and Sanches, D.S. (2018). Sequence motif finder using memetic algorithm. BMC Bioinf. *19*, 4.
18. Kiesel, A., Roth, C., Ge, W., Wess, M., Meier, M., and Söding, J. (2018). The BaMM web server for de-novo motif discovery and regulatory sequence analysis. Nucleic Acids Res. *46*, W215–W220.
19. Hartmann, H., Guthöhrlein, E.W., Siebert, M., Luehr, S., and Söding, J. (2013). P-value-based regulatory motif discovery using positional weight matrices. Genome Res. *23*, 181–194.
20. Wang, L., Chen, J., Wang, C., Uusküla-Reimand, L., Chen, K., Medina-Rivera, A., Young, E.J., Zimmermann, M.T., Yan, H., Sun, Z., et al. (2014). MACE: model based analysis of ChIP-exo. Nucleic Acids Res. *42*, e156.
21. Gupta, S., Stamatoyannopoulos, J.A., Bailey, T.L., and Noble, W.S. (2007). Quantifying similarity between motifs. Genome Biol. *8*, R24–R29.
22. Kiliç, S., White, E.R., Sagitova, D.M., Cornish, J.P., and Erill, I. (2014). CollecTF: a database of experimentally validated transcription factor-binding sites in Bacteria. Nucleic Acids Res. *42*, D156–D160.
23. Kulakovskiy, I.V., Vorontsov, I.E., Yevshin, I.S., Soboleva, A.V., Kasianov, A.S., Ashoor, H., Ba-alawi, W., Bajic, V.B., Medvedeva, Y.A., Kolpakov, F.A., and Makeev, V.J. (2016). HOCOMOCO: expansion and enhancement of the collection of transcription factor binding sites models. Nucleic Acids Res. *44*, D116–D125.
24. McClish, D.K. (1989). Analyzing a portion of the ROC curve. Med. Decis. Making *9*, 190–195.
25. Decker, K.T., Gao, Y., Rychel, K., Al Bulushi, T., Chauhan, S.M., Kim, D., Cho, B.-K., and Palsson, B.O. (2022). proChIPdb: a chromatin immunoprecipitation database for prokaryotic organisms. Nucleic Acids Res. *50*, D1077–D1084.
26. Lai, W.K.M., Mariani, L., Rothschild, G., Smith, E.R., Venters, B.J., Blanda, T.R., Kuntala, P.K., Bocklund, K., Mairose, J., Dweikat, S.N., et al. (2021). A ChIP-exo screen of 887 Protein Capture Reagents Program transcription factor antibodies in human cells. Genome Res. *31*, 1663–1679.
27. Li, Y., Wang, Y., Wang, C., Ma, A., Ma, Q., and Liu, B. (2023). A weighted two-stage sequence alignment framework to identify motifs from ChIP-exo data. Zenodo. https://doi.org/10.5281/zenodo.10436358.
28. Quinlan, A.R., and Hall, I.M. (2010). BEDTools: a flexible suite of utilities for comparing genomic features. Bioinformatics *26*, 841–842.

Patterns, Volume *5*

Supplemental information

A weighted two-stage sequence alignment framework to identify motifs from ChIP-exo data

Yang Li, Yizhong Wang, Cankun Wang, Anjun Ma, Qin Ma, and Bingqiang Liu

1. Note S1: Parameters for benchmarking algorithms

We execute the seven designated motif discovery algorithms using specific options, with values contingent upon the individual experiment. Version numbers for the algorithms are denoted immediately after their respective names.

- BoBro v.2.0:
  ```
  BoBro -i <primary> -l 14 -o 100
  ```
  where <primary> is the file of input sequences.
- Homer v.4.11:
  ```
  homer2 denovo -i <primary> -b <control> -S 100 -len 10 > <outdir>
  ```
  where <outdir> is the directory where Homer will write its results and <control> is the file of background sequences.
- MEME v.5.5.4:
  ```
  meme <primary> -dna -revcomp -minw 8 -maxw 50 -nmotifs 100
  ```
- MFMD v.1.0:
  ```
  java -jar mfmd.jar <primary> 22 0.005
  ```
- PEnGmotif v.1.0.1:
  ```
  PEnGmotif <primary> -o <outdir> --background-sequences <control>
  ```
- STREME v.5.5.4:
  ```
  streme -p <primary> -n <control> -dna -minw 8 -maxw 15 -kmer 3 -text
  ```
- TESA v.1.0:
  ```
  TESA -i <primary> -l 14 -n 1 -c 0.8 -u 0.9 -o 100
  ```
- XXmotif v.1.6:
  ```
  XXmotif <outdir> <primary> --revcomp
  ```

2. Note S2: Alignment of TESA-cov and reference motifs

In each panel, the top two lines display the HOCOMOCO[1] motif used for experimental evaluation. The sequence logos depict the TESA motif (bottom) juxtaposed with the reference motif (top). Above these aligned logos, the Tomtom P-value represents the accuracy of the motif identified by TESA, with a lower value indicating greater similarity to the reference motif.

Fur (*P.aeruginosa*)
5.78e-13

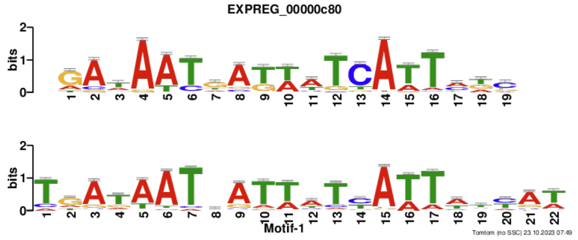

Fur (*A.salmonicida*)
1.74e-11

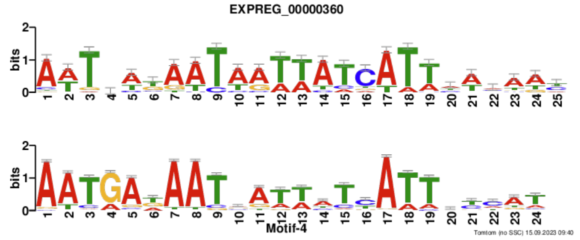

Fur (*C.crescentus*)
2.88e-04

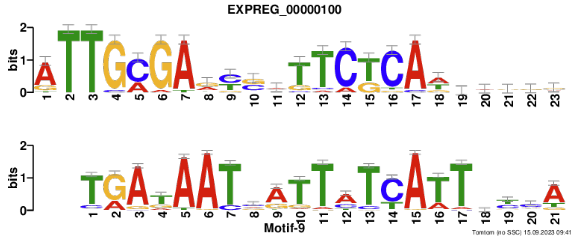

Fur (*N.gonorrhoeae*)
3.03e-09

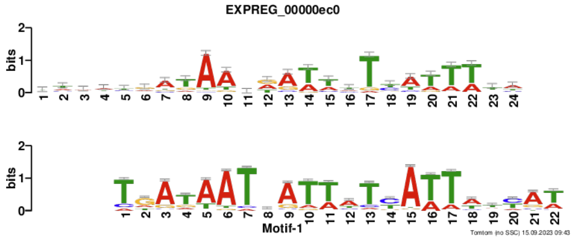

LuxR (*V.campbellii*)
8.82e-04

OxyR (*P.aeruginosa*)
4.81e-04

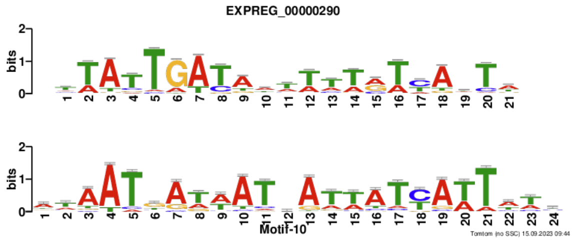

NikR (*H.pylori*)
1.34e-03

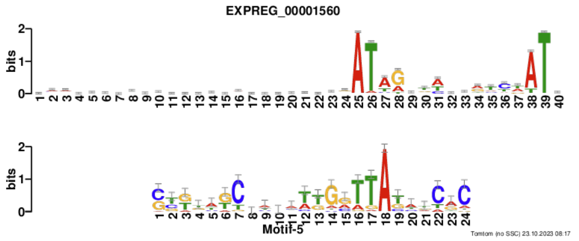

GlxR (*C.glutamicum*)
5.87e-06

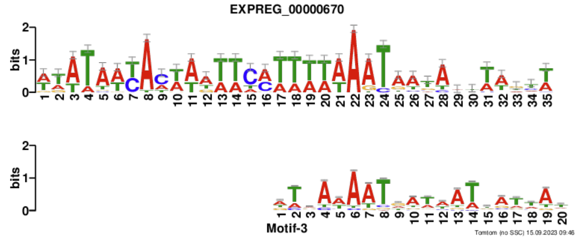

Fur (*P.syringae*)
4.35e-07

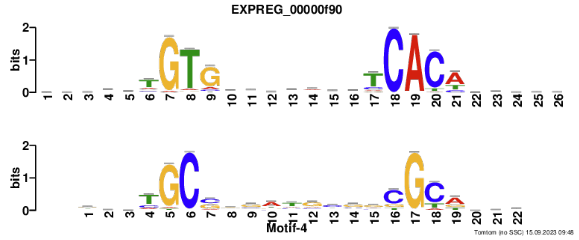

CodY (*L.lactis*)
2.06e-05

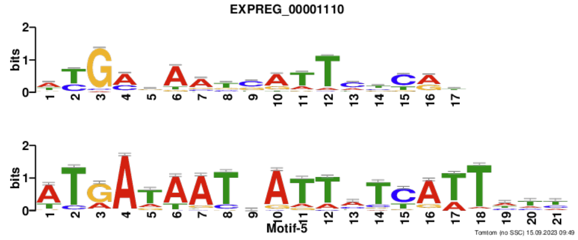

ArgR (*P.aeruginosa*)
8.41e-04

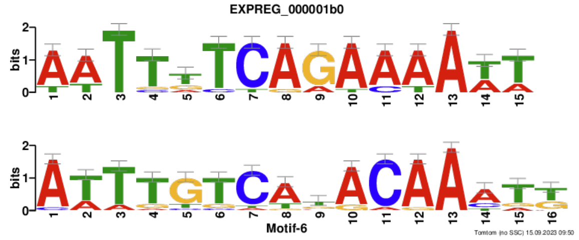

Fur (*B.subtilis*)
8.66e-12

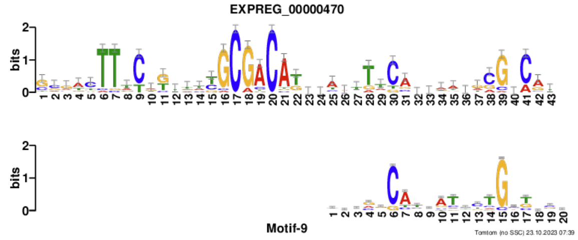

PerR (*B.subtilis*)
1.83e-05

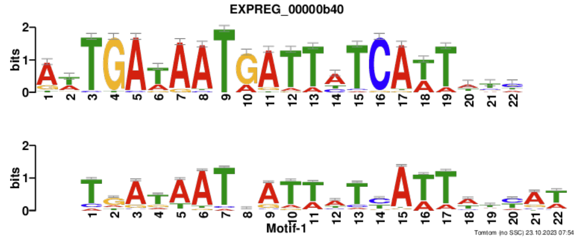

AmrZ (*P.aeruginosa*)
6.04e-04

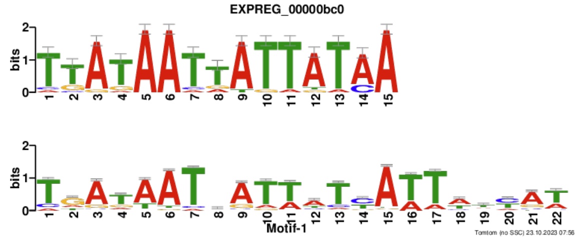

H-NS (*V.cholerae*)
7.33e-06

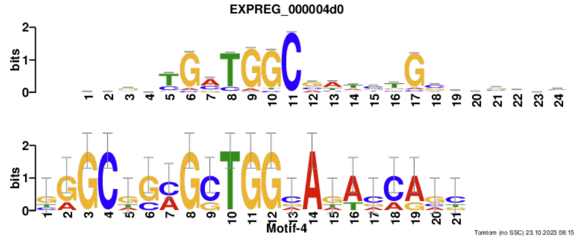

IHF (*P.putida*)
5.27e-06

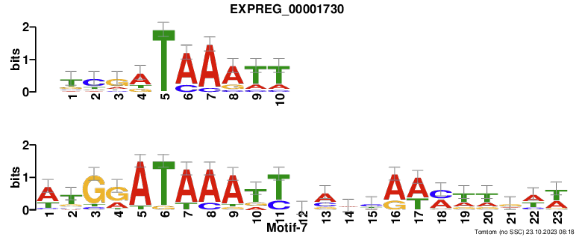

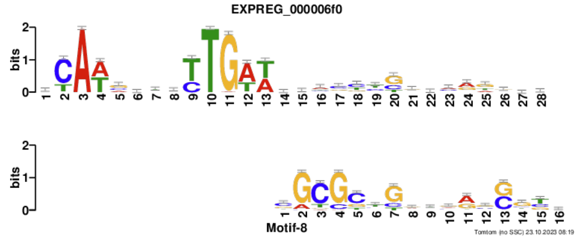

Vfr (*P.aeruginosa*)
1.56e-03

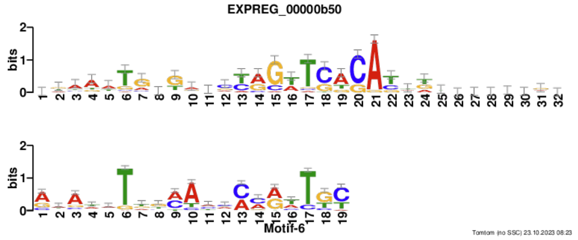

PvdS (*P.aeruginosa*)
2.50e-04

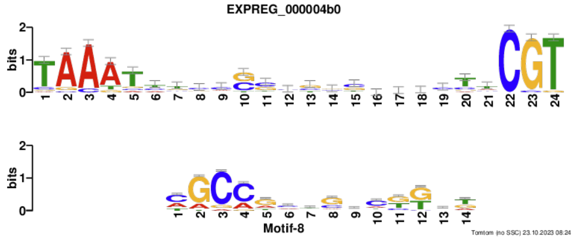

VqsM (*P.aeruginosa*)
4.57e-04

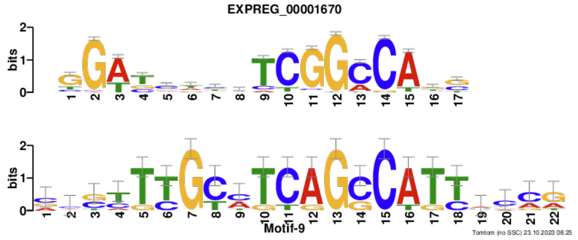

LexA (*M.tuberculosis*)
5.55e-04

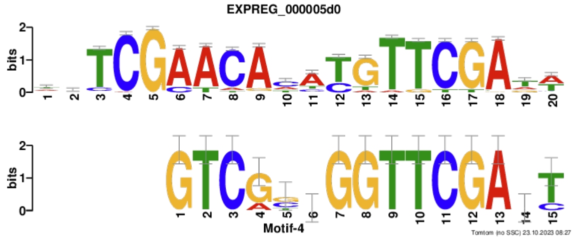

DosR (*M.tuberculosis*)
7.47e-04

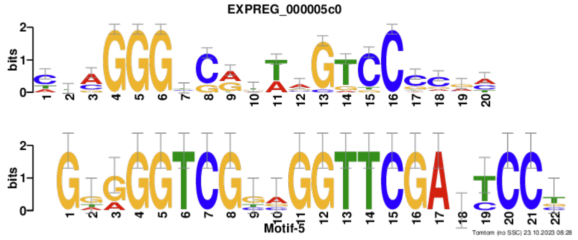

HrpX (*X.oryzae*)
2.63e-04

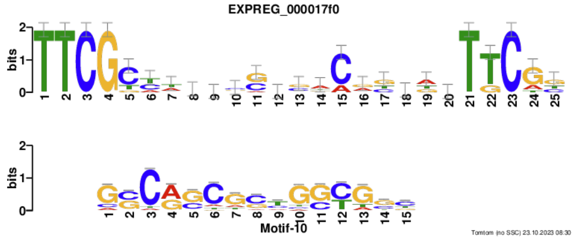

EspR (*M.tuberculosis*)
4.72e-04

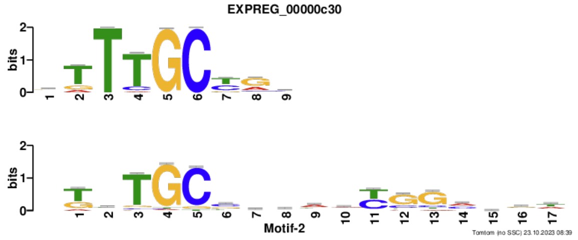

MogR (*L.monocytogenes*)
4.39e-04

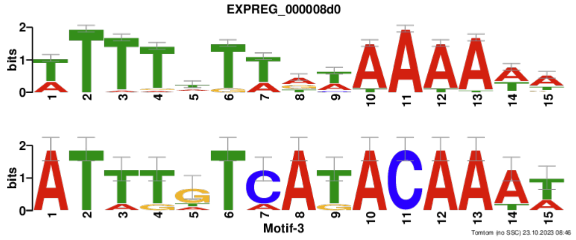

OmpR (*Y.pestis*)
5.18e-04

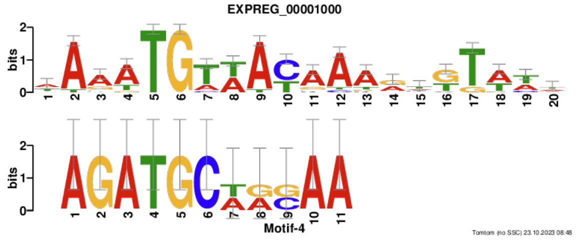

ZN502 (*H. sapiens*)
7.76e-10

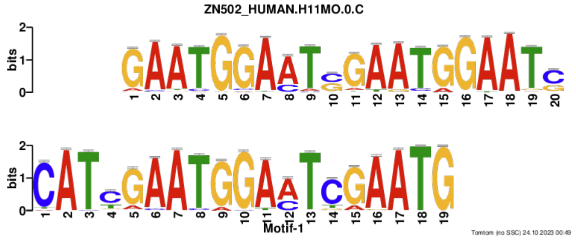

SMCA5 (*H. sapiens*)
4.89e-08

ZN341 (*H. sapiens*)
2.98e-09

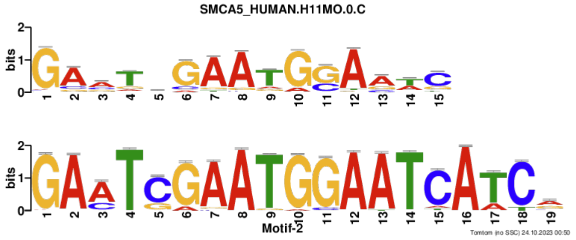

PO2F1 (*H. sapiens*)
1.28e-05

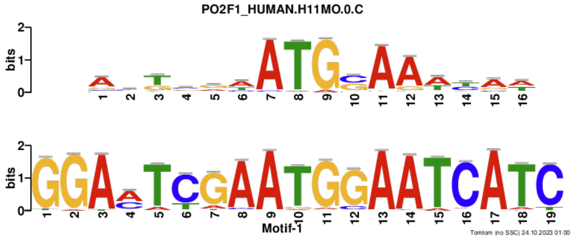

TBX15 (*H. sapiens*)
5.89e-09

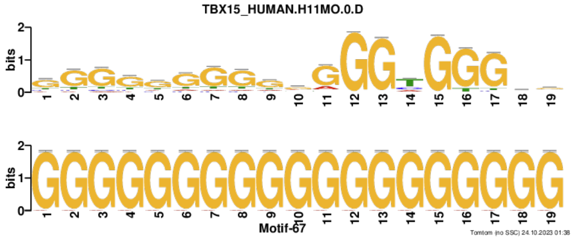

KLF16 (*H. sapiens*)
5.94e-08

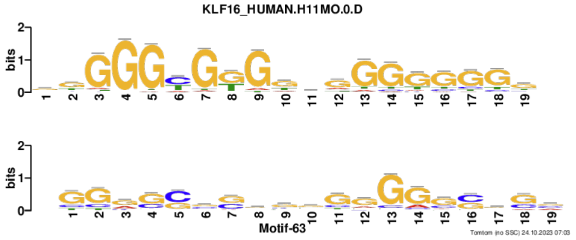

ZNF8 (*H. sapiens*)
4.75e-05

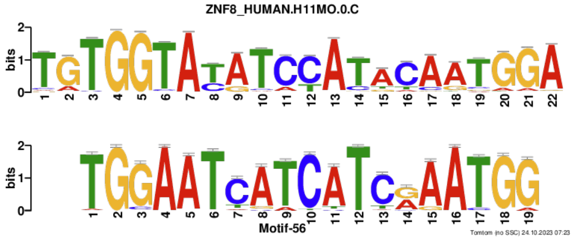

ZN394 (*H. sapiens*)
5.03e-05

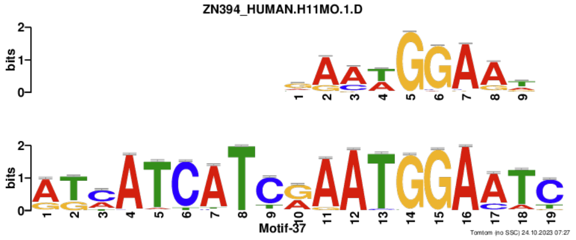

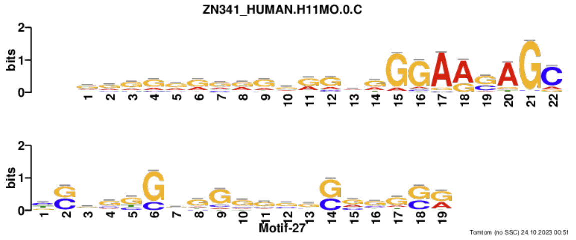

CENPB (*H. sapiens*)
2.08e-07

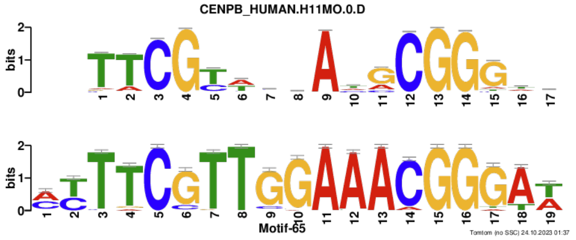

ATF2 (*H. sapiens*)
7.69e-05

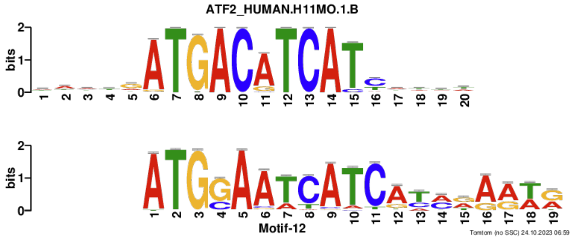

ZN214 (*H. sapiens*)
2.63e-06

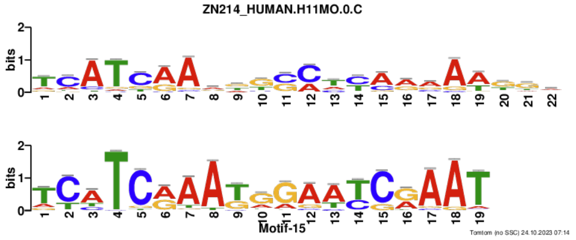

ZN582 (*H. sapiens*)
4.49e-09

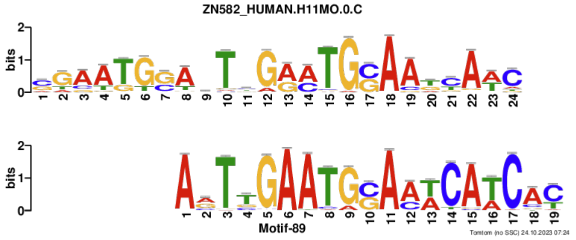

PO3F2 (*H. sapiens*)
1.05e-07

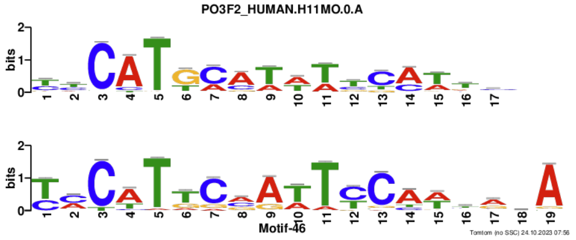

STA5B (*H. sapiens*)
1.05e-04

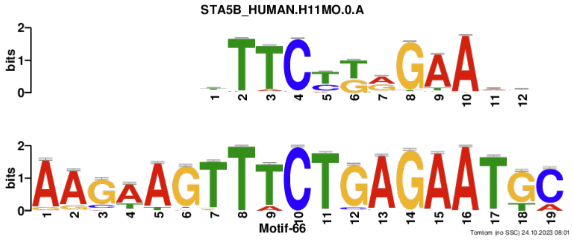

PATZ1 (*H. sapiens*)
1.24e-09

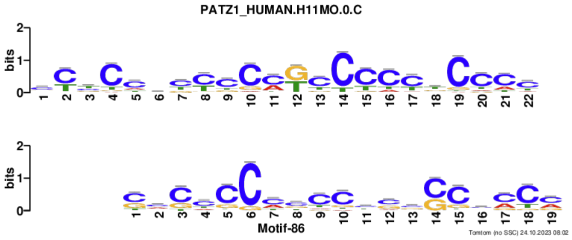

FOXM1 (*H. sapiens*)
5.40e-05

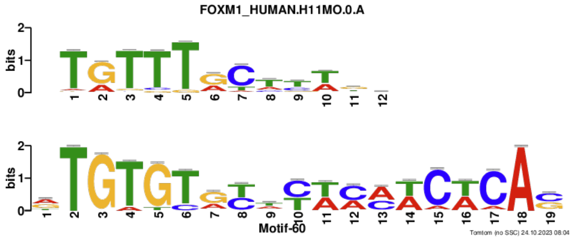

STAT6 (*H. sapiens*)
8.22e-05

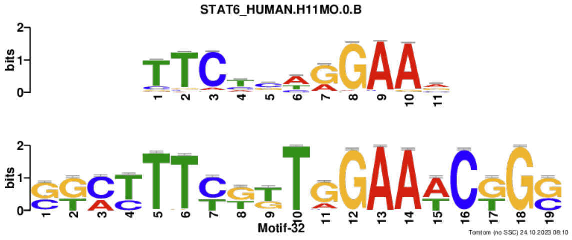

Z354A (*H. sapiens*)
1.89e-05

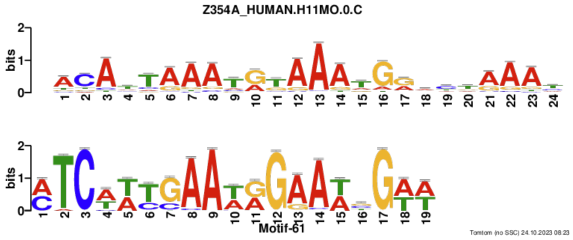

IRF3 (*H. sapiens*)
5.73e-05

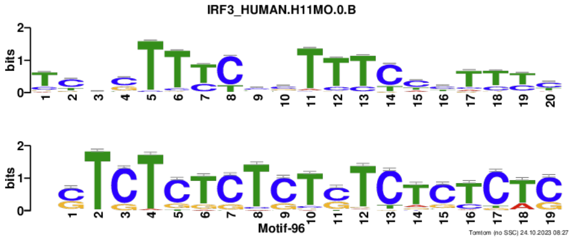

STA5A (*H. sapiens*)
1.75e-05

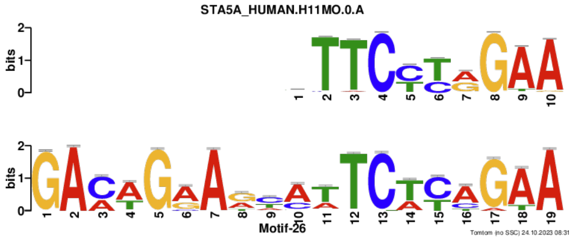

ZN563 (*H. sapiens*)
1.18e-06

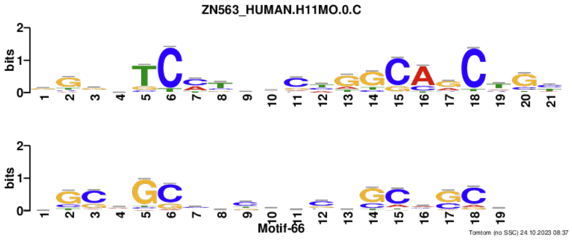

ZN667 (*H. sapiens*)
4.14e-05

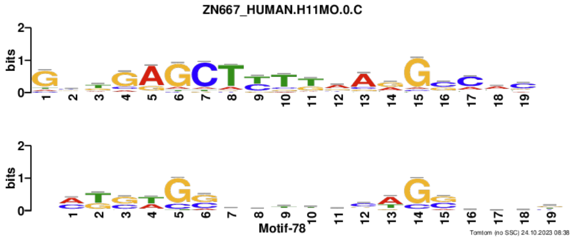

MNX1 (*H. sapiens*)
5.26e-05

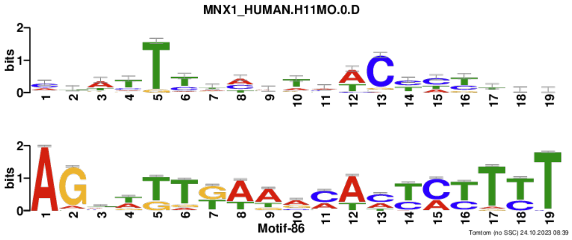

STAT4 (*H. sapiens*)
2.00e-05

SP2 (*H. sapiens*)
4.36e-07

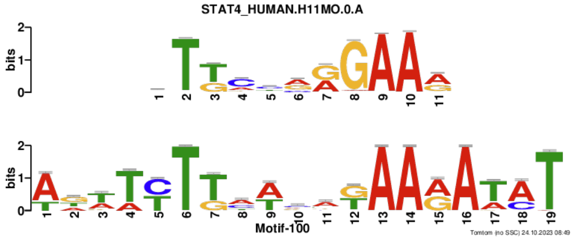

SP3 (*H. sapiens*)
7.25e-07

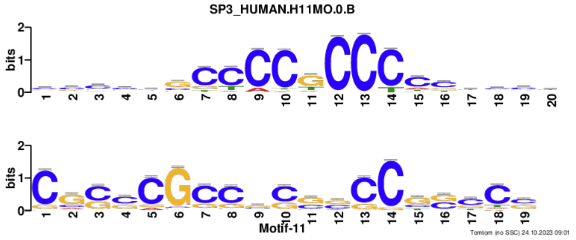

CPEB1 (*H. sapiens*)
1.43e-06

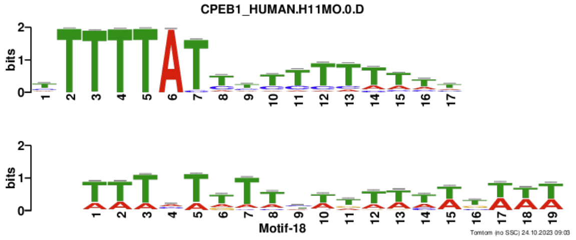

MAZ (*H. sapiens*)
4.14e-07

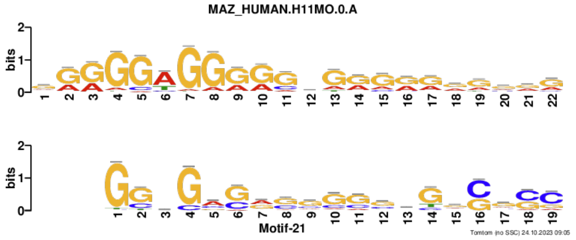

PO3F3 (*H. sapiens*)
4.67e-07

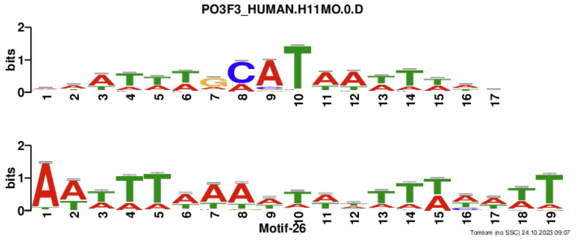

ZN335 (*H. sapiens*)
1.14e-06

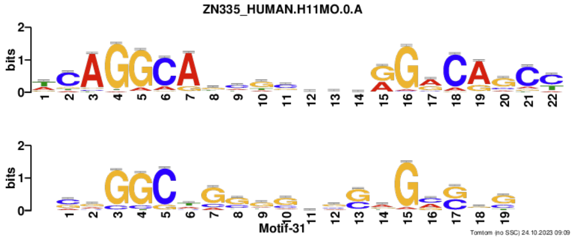

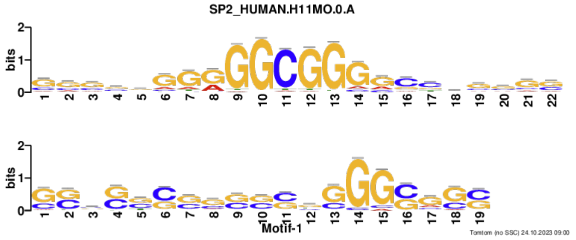

THAP1 (*H. sapiens*)
5.80e-06

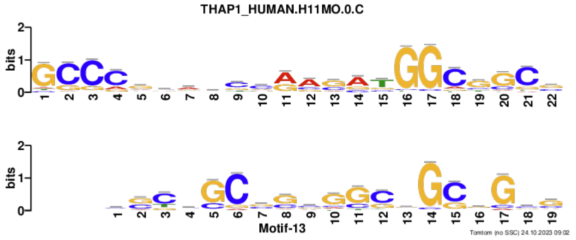

ZFX (*H. sapiens*)
2.44e-05

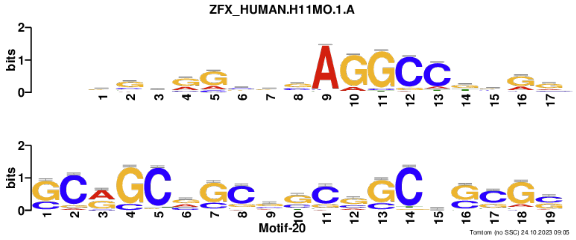

HXC10 (*H. sapiens*)
5.84e-06

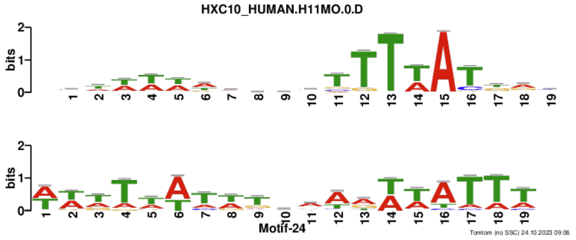

ZN148 (*H. sapiens*)
2.16e-08

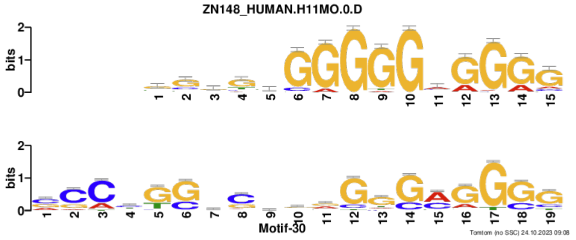

ANDR (*H. sapiens*)
6.36e-06

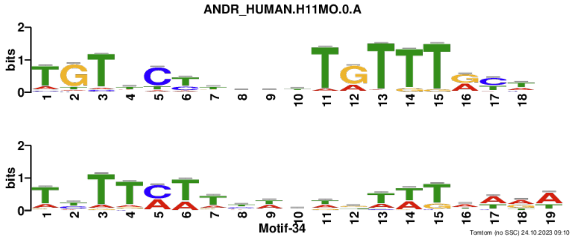

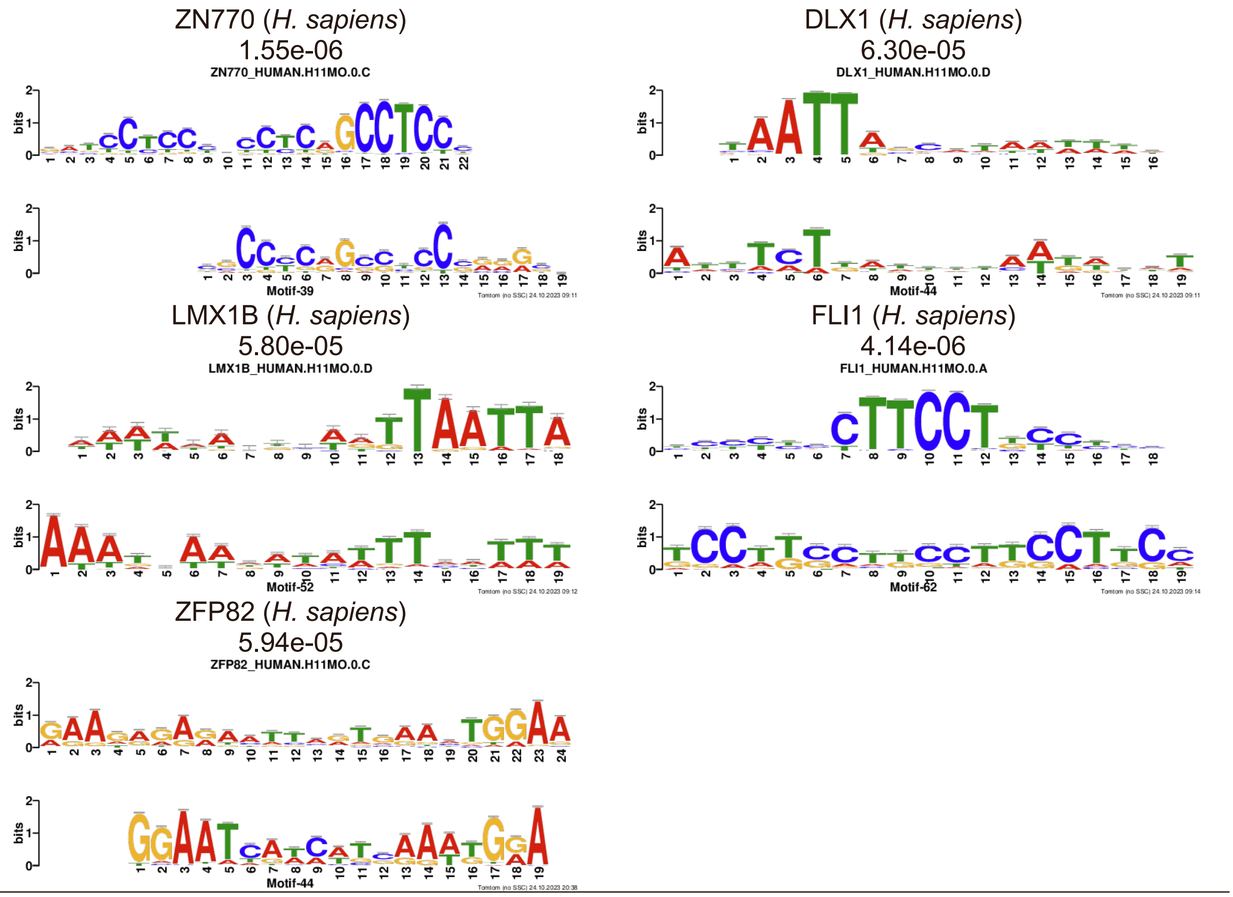

3. Note S3: Data acquisition

The raw sequencing data corresponding to GSE152144[2], in SRA format, were retrieved from the Sequencing Read Archive (SRA) using the accession code PRJNA638411[3]. These SRA files were then converted into FASTQ format utilizing the **fastq-dump** utility from the SRA Toolkit (v.3.0.2)[3]. For the alignment process, we employed Bowtie2 (v.2.4.1)[4] to accurately align the sequencing reads, now in FASTQ format, against the *H. sapiens* genome reference (hg19). This resulted in the generation of Sequence Alignment Map (SAM) files.

In the subsequent step, we converted these SAM files into more compressed Binary Alignment Map (BAM) files using SAMtools (v.1.16.1)[5] and performed a sorting procedure. Peak calling was conducted to identify narrow peaks, which were subsequently represented in Browser Extensible Data (BED) format. This was achieved by executing the **callpeak** function in MACS2 (v.2.2.9.1)[6].

For the benchmarking process, we required sequences to act as positive controls. Therefore, we employed the **getfasta** utility from BEDTools (version 2.29.1) to extract the sequences corresponding to these narrow peaks, resulting in their representation in FASTA format. These sequences served as the positive sequences for our benchmarking analysis.

For each positive sequence identified, we employed the R package Biostrings (v.2.66.0) to meticulously extract a random sequence of equivalent length from the *H. sapiens* genome, thereby generating a corresponding set of negative sequences. These positive and negative sequences were subsequently amalgamated to formulate the input dataset essential for rigorous benchmarking procedures.

Analytical tools such as Homer[7] and STREME[8] necessitate an auxiliary set of sequences to serve as a background reference. To fulfill this requirement, we again harnessed the capabilities of Biostrings, enabling

us to randomly curate a collection of sequences from the *H. sapiens* genome. Each sequence within this compilation mirrored the lengths of those found in our input dataset, ensuring consistency in our comparative analysis for benchmarking.

Furthermore, proChIPdb[9] conveniently furnishes us with narrow peaks, delineated in BED format, for each pertinent dataset. This provision allows us to directly incorporate these datasets into our benchmarking framework, streamlining the analysis process.

4. Note S4: Data preparation

We present TESA, a novel composite file format designed to integrate DNA sequences with their associated sequencing coverages. Constructing the TESA format necessitates a reference genome in FASTA format, narrow peaks in BED format, and sequencing coverage files in bigWig format. Initially, narrow peaks are alphabetically organized and uniformly adjusted to a standard length (typically 100 bp) using the **sort** and **awk** Shell utilities. Thereafter, the **bigWigToBedGraph** function[10] transitions the sequencing coverage files to the bedGraph format, enhancing versatility. Utilizing BEDTools, the **complement** function then standardizes sequencing coverages for the reverse complementary sequences of the narrow peaks. Conclusively, **getfasta** transforms narrow peaks from BED to FASTA format. These sequences, combined with bidirectional sequencing coverages, constitute the TESA file format.

5. Note S5: Calculation of weights in two-stage alignment

TESA employs a two-stage alignment strategy utilizing a matrix, M^h, of dimensions $2m \times n$. In M^h, the rows depict both the input sequences (odd rows) and their reverse complements (even rows). The columns mark the starting positions of segments, each of length l, on the sequences. Matrix entries encapsulate the normalized sequencing coverages. For segments s_{ij} from sequence s_i and s_{pq} from sequence s_p with k identical positions, TESA leverages the binomial distribution, denoted in **Equation 1**, to assess the statistical significance of their sequence similarity,

$$f(s_{ij}, s_{pq}) = -\lg\left(\sum_{k}^{l} B(l, k, 0.25)\right). \qquad \text{(Equation 1)}$$

If $B(l, k, 0.25) > 0.01$, TESA sets $f(s_{ij}, s_{pq}) = 0$. Then, TESA calculates the weight of the segment pair s_{ij} and s_{pq} by incorporating the sequencing coverage of both segments, as described by

$$f'(s_{ij}, s_{pq}) = f(s_{ij}, s_{pq}) \times (M_{ij}^h + M_{pq}^h). \qquad \text{(Equation 2)}$$

If $f(s_{ij}, s_{pq})$ surpasses a preset threshold (defaulted to 3) or if $f'(s_{ij}, s_{pq})$ is among the top α alignments (defaulted to 5) for all l-segment pairings, TESA augments both M_{ij}^1 and M_{pq}^1 by β. In TESA, the value of β is ascertained by evaluating the antecedent dinucleotides of s_{ij} and s_{pq}. When both segments share an identical preceding dinucleotide, β is set to 0.5; otherwise, it assumes a value of 1.0.

REFERENCES

1. Kulakovskiy, I.V., Vorontsov, I.E., Yevshin, I.S., Soboleva, A.V., Kasianov, A.S., Ashoor, H., Ba-alawi, W., Bajic, V.B., Medvedeva, Y.A., Kolpakov, F.A., and Makeev, V.J. (2015). HOCOMOCO: expansion and enhancement of the collection of transcription factor binding sites models. *Nucleic Acids Res 44*, D116-D125. 10.1093/nar/gkv1249.
2. Lai, W.K.M., Mariani, L., Rothschild, G., Smith, E.R., Venters, B.J., Blanda, T.R., Kuntala, P.K., Bocklund, K., Mairose, J., Dweikat, S.N., et al. (2021). A ChIP-exo screen of 887 Protein Capture Reagents Program transcription factor antibodies in human cells. *Genome Res 31*, 1663-1679. 10.1101/gr.275472.121.
3. Katz, K., Shutov, O., Lapoint, R., Kimelman, M., Brister, J R., and O'Sullivan, C. (2021). The Sequence Read Archive: a decade more of explosive growth. *Nucleic Acids Res 50*, D387-D390. 10.1093/nar/gkab1053.
4. Langmead, B., and Salzberg, S.L. (2012). Fast gapped-read alignment with Bowtie 2. *Nat Methods 9*, 357-359. 10.1038/nmeth.1923.

5. Danecek, P., Bonfield, J.K., Liddle, J., Marshall, J., Ohan, V., Pollard, M.O., Whitwham, A., Keane, T., McCarthy, S.A., Davies, R.M., and Li, H. (2021). Twelve years of SAMtools and BCFtools. *GigaScience 10*. 10.1093/gigascience/giab008.
6. Zhang, Y., Liu, T., Meyer, C.A., Eeckhoute, J., Johnson, D.S., Bernstein, B.E., Nusbaum, C., Myers, R.M., Brown, M., Li, W., and Liu, X.S. (2008). Model-based Analysis of ChIP-Seq (MACS). *Genome Biol 9*, R137. 10.1186/gb-2008-9-9-r137.
7. Duttke, S.H., Chang, M.W., Heinz, S., and Benner, C. (2019). Identification and dynamic quantification of regulatory elements using total RNA. *Genome Res 29*, 1836-1846. 10.1101/gr.253492.119.
8. Bailey, T.L. (2021). STREME: accurate and versatile sequence motif discovery. *Bioinformatics 37*, 2834-2840. 10.1093/bioinformatics/btab203.
9. Decker, K.T., Gao, Y., Rychel, K., Al Bulushi, T., Chauhan, Siddharth M., Kim, D., Cho, B.-K., and Palsson, Bernhard O. (2021). proChIPdb: a chromatin immunoprecipitation database for prokaryotic organisms. *Nucleic Acids Res 50*, D1077-D1084. 10.1093/nar/gkab1043.
10. Kent, W.J., Sugnet, C.W., Furey, T.S., Roskin, K.M., Pringle, T.H., Zahler, A.M., and Haussler, D. (2002). The human genome browser at UCSC. *Genome Res 12*, 996-1006. 10.1101/gr.229102.
